# Electrostatically-guided inhibition of Curli amyloid nucleation by the CsgC-like family of chaperones

**DOI:** 10.1038/srep24656

**Published:** 2016-04-21

**Authors:** Jonathan D. Taylor, William J. Hawthorne, Joanne Lo, Alexander Dear, Neha Jain, Georg Meisl, Maria Andreasen, Catherine Fletcher, Marion Koch, Nicholas Darvill, Nicola Scull, Andrés Escalera-Maurer, Lea Sefer, Rosemary Wenman, Sebastian Lambert, Jisoo Jean, Yingqi Xu, Benjamin Turner, Sergei G. Kazarian, Matthew R. Chapman, Doryen Bubeck, Alfonso de Simone, Tuomas P. J. Knowles, Steve J. Matthews

**Affiliations:** 1 Department of Life Sciences, Imperial College London, London, SW7 2AZ, UK; 2 Department of Chemistry, University of Cambridge, Lensfield Road, Cambridge CB2 1EW, UK; 3 Department of Molecular, Cellular, and Developmental Biology, University of Michigan, Ann Arbor, MI 48109, USA; 4 Department of Chemical Engineering, Imperial College London, London, SW7 2AZ, UK

## Abstract

Polypeptide aggregation into amyloid is linked with several debilitating human diseases. Despite the inherent risk of aggregation-induced cytotoxicity, bacteria control the export of amyloid-prone subunits and assemble adhesive amyloid fibres during biofilm formation. An *Escherichia* protein, CsgC potently inhibits amyloid formation of curli amyloid proteins. Here we unlock its mechanism of action, and show that CsgC strongly inhibits primary nucleation via electrostatically-guided molecular encounters, which expands the conformational distribution of disordered curli subunits. This delays the formation of higher order intermediates and maintains amyloidogenic subunits in a secretion-competent form. New structural insight also reveal that CsgC is part of diverse family of bacterial amyloid inhibitors. Curli assembly is therefore not only arrested in the periplasm, but the preservation of conformational flexibility also enables efficient secretion to the cell surface. Understanding how bacteria safely handle amyloidogenic polypeptides contribute towards efforts to control aggregation in disease-causing amyloids and amyloid-based biotechnological applications.

The formation of amyloid fibres by proteins is widely-known to endanger organism health^1^. Yet many organisms are able to safely direct particular proteins or peptides into fibrous amyloid structures, and thus benefit from the self-assembling, stable structure^2^. Such ‘functional amyloids’ are found in prokaryotes and eukaryotes where they play diverse roles such as protein storage^3^, extracellular matrices^4^,^5^,^6^, epigenetic inheritance^7^, silk formation^8^, melanin production^9^, and bacteriocidal immune responses^10^. By controlling early and intermediate stages of amyloid formation, organisms are able to avoid any undesirable side-effects from amyloidogenesis. The study of functional amyloid systems will therefore yield insights into treatment of disease-causing amyloidoses and aid in the development of bio-materials and anti-biofilm strategies^11^.

The curli system of gram-negative bacteria is one of the best-understood functional amyloids^12^,^13^. Curli fibres are surface-attached, extracellular appendages that contribute towards surface adhesion and biofilm formation^5^. The main fibre component, CsgA, is thought to form a compact, parallel β-helix structure that aggregates into amyloid fibres, however little is known regarding the intermediate steps *en route*^14^. Curli fibres are distinct from other bacterial fimbriae in that subunit folding and multimerisation occurs extracellularly^15^. CsgA subunits are efficiently exported by the CsgEG complex where CsgA assembles into fibres that are attached to the cell surface^16^,^17^. The bacterium employs two proteins – CsgF and CsgB – to control the location and structural templating, respectively, of this nucleation event. Thus a picture emerges of a cascade of interactions that efficiently steers aggregation-prone CsgA monomers into an amyloid fibre whilst avoiding toxicity.

In order to maximise secretion efficiency, CsgA should be maintained in a relatively high-energy folding state until it arrives outside the cell. Recently, we reported that CsgC potently inhibits the formation of amyloid fibres by CsgA^18^. CsgC is not irreversibly incorporated into fibres and is effective at extreme substoichiometric ratios (i.e. even in a 10^2^–10^3^ molar excess of CsgA). Discouraging inappropriate amyloid intermediates from forming in the periplasm would not only benefit secretion but also cell vitality as early oligomeric species are often cytotoxic^19^. Thus dealing with oligomers – either by reconfiguring, dissociating, or capping them – could be a way by which CsgC efficiently inhibits amyloid formation. In this report we provide new insight into the mechanism of CsgC inhibition. We extend this family of amyloid inhibitors and identify CsgH as a new member despite limited sequence homology. We also present kinetic, biochemical, and biophysical analyses and unveil the mechanism by which CsgC and CsgH affect amyloid formation.

## Results

### CsgH and CsgC adopt the same structural fold

Until recently the curli operon was only known to be present amongst *Enterobacteriales*, eminently within *Escherichia* and *Salmonella.* A search of sequence databases revealed that curli-like operons occur throughout several bacterial kingdoms, albeit with a greater degree of genetic diversity^12^. Outside of γ-proteobacteria, the standard twin operons consisting of *csgDEFG* and *csgBAC* are generally unified into a single, minimal operon that lacks clear homologues of *csgC* and the master biofilm regulator *csgD*. An additional gene was also identified within α-proteobacteria, termed *csgH*, which bears little sequence homology to other curli genes^12^. However, when considering likely secondary structure, putative disulphides, and genetic location (adjacent to the subunit genes), CsgH display some resemblance to CsgC (Fig. S1).

**Figure 1:**
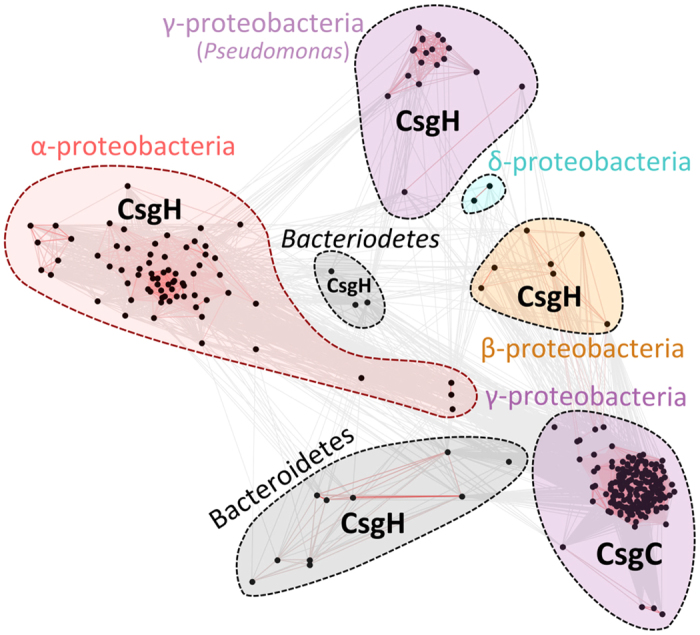
Phylogenetic distribution of CsgC- and CsgH-like sequences. Distinct families of CsgC- and CsgH-like sequences occur across gram-negative bacterial phyla. The CsgC group is tightly-defined whereas CsgH can be split into several sub-types. The red lines connecting dots represent relatively-close sequence homology.

To shed more light on this intriguing similarity used sequential iterations of PSI-BLAST to obtain 299 unique CsgC/CsgH sequences. These homologues were clustered in two dimensions according to pair-wise sequence similarity using CLANS (Fig. 1). We define a protein as a CsgC-like or CsgH-like according to its disulphide bonding pattern (i.e. CsgC homologues have a C × C motif whereas the cysteines are distant in sequence within CsgH homologues). CsgH was initially discovered in α-proteobacteria, however there are also distinct sub-families in γ-, β-, and δ-proteobacteria as well as *Bacteroidetes*. The detectable sequence similarity between CsgH and CsgC suggests that they may share the same ancestral origins. It is also worth noting that CsgH sequences are far more diverse than CsgC, which likely reflects the nature of its substrate, CsgA. CsgA sequences are extremely varied in strains carrying CsgH-like genes, with variations in operon organisation and numbers of amyloid repeats and even the number of curli subunits, and this is particularly notable for the *Bacteriodetes*^12^.

**Figure 2:**
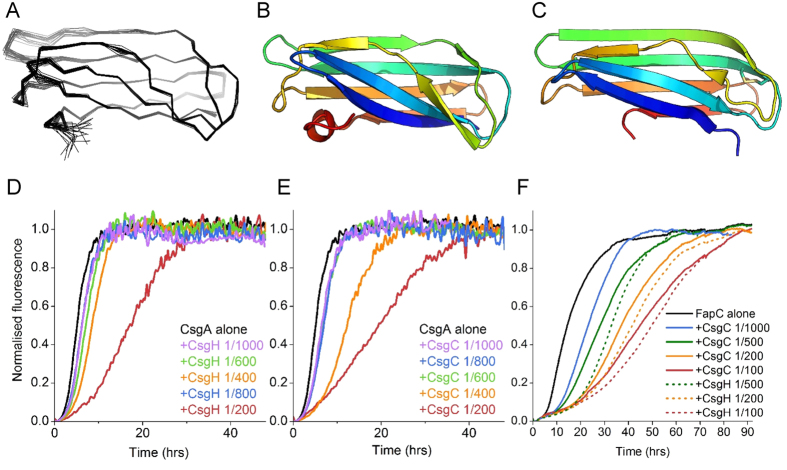
CsgH is a structural and functional homologue of CsgC. (**A**) Ensemble of 20 lowest-energy structures calculated for CsgH (PDB 2N59). (**B**) Cartoon structure of the lowest-energy CsgH structure coloured according to the rainbow from N- (blue) to C-terminus (red). (**C**) Crystal structure of CsgC (PDB 2Y2Y) shown for comparison. Addition of (**D**) CsgH or (**E**) CsgC, respectively, at a range of substoichiometric molar ratios leads to a dose-dependent inhibition in the kinetic profile of CsgA amyloid formation within the ThT assay. (**F**) Addition of CsgC (solid lines) or CsgH (dashed lines) at various substoichiometric ratios results in a dose-dependent inhibition of FapC amyloid formation.

Since CsgC and CsgH display significantly different sequences, we elucidated the structure of CsgH using nuclear magnetic resonance (Fig. 2A–C and Table 1). The structure reveals seven β-strands arranged in an identical configuration to CsgC. One notable difference is the location of the disulphide bond, which staples the N- and C-terminal strands together in CsgH (Fig. S1). Structural alignment between CsgH and CsgC (rmsd = 2.6 Å) underlines their similarity despite the level of sequence identity being below 20%. In fact, the most similar structure to CsgH within the current PDB archive is CsgC.

### CsgH is a functional homologue of CsgC

Conservation of structure between CsgC and CsgH may indicate that they utilise this particular fold for the same function, or it could reflect the stability of this ancient protein fold. The fact that no operons exist containing both *csgC* and *csgH* is also supportive of functional homology. Previous studies have shown that CsgC is a potent inhibitor of CsgA amyloid formation^18^. We therefore tested whether CsgH has analogous inhibitory properties using the Thioflavin T (ThT) fluorescence assay to monitor its effect on CsgA amyloid formation. Indeed, recombinant CsgH from *Rhodopseudomonas palustris* was able to inhibit amyloid formation of *E. coli* CsgA in a dose-dependent manner at potencies similar to CsgC (Fig. 2D–E).

Given that CsgC and CsgH can inhibit the fibrillation of amyloidogenic proteins outside of their natural curli operon we tested their effectiveness against a unrelated bacterial amyloid protein: FapC from *Pseudomonas aeruginosa*^4^. Using the ThT assay we mixed samples of recombinant FapC with CsgC (or CsgH) at a range of substoichiometric molar ratios. Remarkably both proteins were potent inhibitors of FapC amyloid formation, with CsgH being slightly more effective than CsgC (Fig. 2F).

### CsgC delays the appearance of fibrillar structures *in vitro*

**Figure 3:**
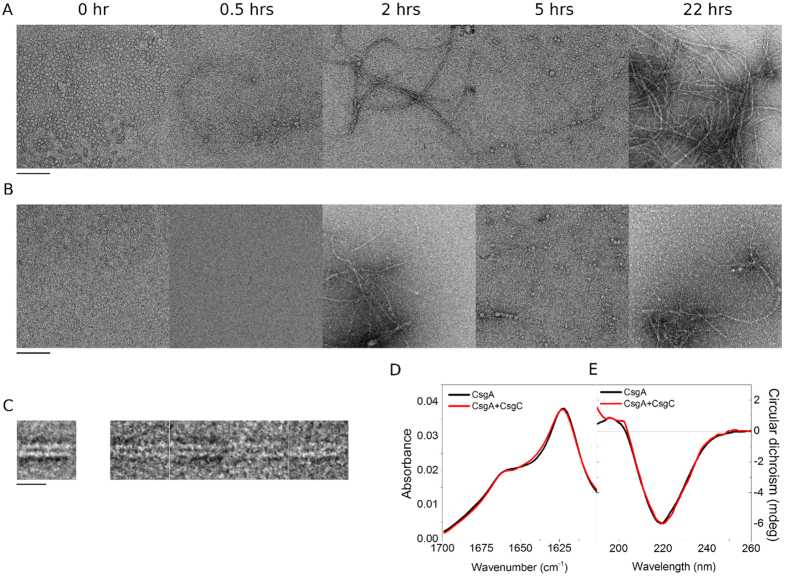
Negative stain EM images documenting a time-course for nucleating fibres. Representative images from (**A**) CsgA and (**B**) CsgA + CsgC fibre formation are shown. CsgC was added at a substoichiometric ratio of 1:200. Time points for 0, 0.5, 2, 5 and 22 hours are shown (see additional. Scale bar = 200 nm. Additional examples for each time point are shown in Figs S2 and S4. (**C**) 2D Class average and corresponding raw images for CsgA double filament architecture. Scale bar, 16 nm. Panels (**D,E**) show ATR-FTIR and CD spectra, respectively, of CsgA fibres grown for 24 hrs in the absence or presence of CsgC (200:1 molar ratio).

To determine at what stage in amyloid formation CsgC functions, we explored negative-stain electron microscopy to provide a qualitative visualization of CsgA aggregation. Images were recorded at 0, 0.5, 1, 2, 3, 5 and 22 hours post-purification (Fig. 3A; Fig. S2). CsgA fibrils are observed already at the 0.5 hour time point in the absence of CsgC. Extended CsgA fibres at later time-points exhibit a high degree of plasticity (Fig. 3A). However, constituent double filaments were sufficiently thin, linear and homogenous (~10 nm diameter) to be studied by single-particle analysis. Short segments of double filaments were extracted, aligned and classified. In contrast to the helical architecture observed for RepA oligomers^20^ and amyloid β-protein protofibrils^21^, 2D class averages of CsgA filaments showed stacked parallel lines lacking a clear helical structure (Fig. 3C). Filaments are seen exhibiting a twist, however the repeat unit was too large and variable for helical parameters to be assigned.

To give a visual appreciation of the effect of CsgC on CsgA fibre formation, an equivalent time course was performed in parallel in the presence of the inhibitor at a substoichiometric molar ratio of 1:200 (Figs 3B and S2). In contrast to CsgA alone, early time-points showed fewer higher-order assemblies. The presence of small fibre networks became prevalent only at around the 2 hour time point and later. These data suggest that CsgC interacts either with bulk, monomeric CsgA to inhibit the subsequent formation of higher order species, or with intermediates on the pathway to the formation of the oligomers. Single-particle analysis of double filaments formed by CsgA in the presence of CsgC did not reveal any structural differences compared to those formed by CsgA alone (data not shown). In support of this, we obtained both CD and ATR-FTIR spectra of CsgA amyloid fibres formed in the presence or absence of CsgC and observed no significant difference between the two sets of spectra (Fig. 3D,E).

### Chemical kinetics reveals CsgC inhibits primary nucleation and elongation of CsgA

We further explored the mechanism of CsgC inhibition of CsgA amyloid formation by analysing the kinetics of CsgA fibril formation in the presence and absence of CsgC. The kinetics of ordered amyloid aggregation reactions can be described mathematically by considering distinct steps that lead to formation of mature fibres from monomeric subunits; nucleation, elongation and, where relevant, secondary nucleation and fragmentation^22^. Different integrated rate laws describing the kinetics are obtained, dependent upon the presence or absence of each of these steps in the aggregation mechanism, as well as their possible saturation. These can then each be fitted globally to multiple ThT fluorescence curves for aggregation reactions, using our data analysis platform, AmyloFit^22^. The quality of these fits are compared, and if only one mechanism gives an accurate fit, then it is likely the correct one. The fits to the integrated rate law describing the correct mechanism will yield the monomer dependence and rate constants of the individual microscopic processes.

In the case of CsgA in isolation, unseeded aggregation experiments monitored by ThT fluorescence were performed. These data are well-described by a model that includes primary nucleation and elongation, but excludes secondary nucleation and fragmentation (see Supplementary Methods and Fig. S10). This provides strong evidence that secondary nucleation and fragmentation do not occur in CsgA aggregation.

**Figure 4:**
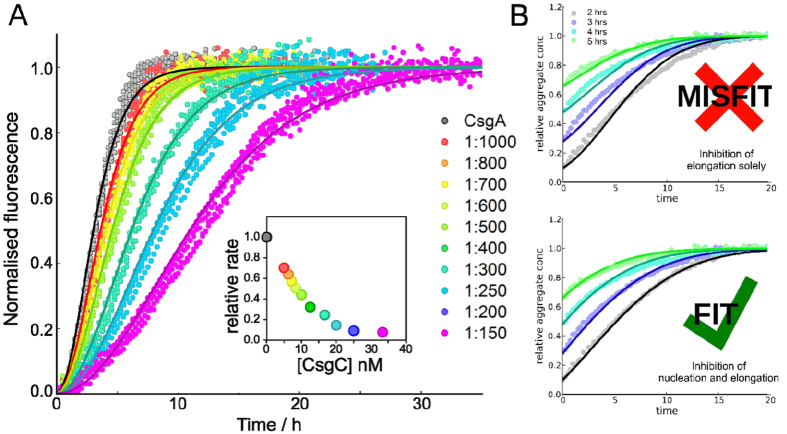
Kinetic analysis of the effect of CsgC on CsgA amyloid formation. (**A**) Addition of CsgC at a range of substoichiometric ratios results in progressive inhibition of CsgA, which was fitted to a nucleation-elongation model [cite Nat Prot]. The change in k + kn with inhibitor concentation is shown inset. (**B**) Addition of CsgC 1:400 at timepoints indicated by legend (in hours). The series truncated at these timepoints can be treated as seeded assays and fitted to a seeded nucleation-elongation model. This allows for the relative contributions of inhibition of nucleation and of elongation to the overall inhibition of k + kn to be separated. This clearly demonstrates a primary inhibitory effect on nucleation with a smaller effect on elongation.

Interestingly, we find that data from unseeded CsgA + CsgC experiments can be fitted to the same model, which shows that CsgC has a perturbative effect on CsgA aggregation, and does not change the underlying mechanism of aggregation. It furthermore suggests that CsgC is not depleted significantly during aggregation, i.e. its binding to aggregating CsgA species is reversible. We do, however, obtain different values of the combined *k*_*+*_*k*_*n*_ nucleation-and-elongation rate constant at each CsgA/ CsgC ratio, which becomes increasingly inhibited as the relative concentration of CsgC increases (Fig. 4A).

In order to separate the relative effect of inhibition on nucleation and on elongation, additional experiments were performed in which CsgC was added to an aggregating CsgA reaction at various intermediate timepoints (Fig. 4B). The mathematical form of the kinetic model was then simplified, using the fact that primary nucleation is first-order in CsgA concentration under the experimental conditions used. A further mathematical analysis of the simplified model revealed a way to analyse these experiments to calculate accurate separated inhibition factors for nucleation and for elongation (see Supplementary Methods for full details). These data analyses revealed that CsgC induces a reduction in both the primary nucleation rate and the elongation rate (Fig. 4B, inset). At a CsgC:CsgA ratio of 1:400 the nucleation rate is reduced by a factor of approximately 2–4 times whereas elongation inhibition is approximately 1.2–2 times. This result is consistent with EM observations in which the appearance of fibrillar structures is delayed by CsgC. Considering the low molar ratio of CsgC used, we therefore conclude that CsgC perturbs CsgA nucleation by inhibiting the formation of some low-concentration intermediate species along the aggregation pathway.

### CsgC affects the conformational distribution CsgA within early, disordered ensembles

In combination with previous results^18^,^23^, our findings suggest that CsgC interacts transiently with CsgA at an early point in its transition to amyloid. We investigated the general structure of CsgA during these early stages, and establish what differences are induced by CsgC. Firstly, we compared ^1^H-NMR spectra of freshly-purified CsgA in the absence or presence of CsgC (200:1 molar ratio). As shown previously, CsgC does not induce any substantial folding of CsgA, however it seems to on the have a small effect average, disordered ensemble structure as evidenced by small peak shifts and sharpening of their linewidths (Fig. S3A).

Next we used ATR-FTIR to discover if CsgC influences the secondary structure profile of CsgA. Using freshly-purified samples of 40 uM CsgA and a 1:200 molar ratio of CsgC:CsgA we collected Amide-I band spectra (Fig. S3B). One explanation for the ATR-FTIR data is that CsgC induces minor alterations in the average CsgA structure as the broad peak centred at ~1650 cm^−1^ representing disordered polypeptide is shifted in the CsgA + CsgC sample. Although this scenario consistent with the NMR data and these data were taken at a very early time point, there may also be a contribution to these ATR-FTIR spectra from oligomeric species with β structure, which would be lower when CsgC is present.

Finally, we explored the overall hydrodynamic state of CsgA at its earliest stages post-purification. Since we postulate that CsgC inhibits nucleation by diverting CsgA from aggregation-prone conformations, we might expect purified CsgA to be more monodisperse in the presence of CsgC. We used SEC-MALS to test this idea (Fig. S3C). During gel filtration, the main CsgA peak displayed an initial shoulder, for which the molecular weight calculation reports the presence of low-population species larger than a monomer. Conversely, when inhibited by CsgC, CsgA eluted as a more monodisperse, monomeric species. We also used pulsed field gradient (PFG)-NMR to measure the diffusion constant of CsgA in the absence and presence of CsgC (at a 200:1 molar ratio). When mixed with CsgC, the self-diffusion coefficient (*D*) of CsgA 1.13 × 10^−10^ m^2^/s. In isolation, this value decreased slightly to 1.03 × 10^−10^ m^2^/s. This trend is consistent with the above findings as, in the presence of CsgC, bulk CsgA diffuses faster, i.e. as more freely diffusing species.

### Electrostatic effects dominate CsgA-CsgC interactions

2D ^1^H-^15^N NMR spectra of 20 μM CsgC or CsgH in the presence of a 3-fold excess of CsgA display a subtle chemical shift and linewidth perturbations for a few specific peaks, which likely indicates a weak and low population interaction with CsgA (Fig. S4). Although these shifts do not group tightly into a binding surface, they decorate the perimeter of the β4-β5 edge of CsgH. As the NMR titration required much higher CsgA concentrations and inhibitor ratios than normally used in amyloid formation assays, and coupled with the sensitivity of amyloid kinetics to monomer concentrations and liquid interfaces, we reverted to site-directed mutagenesis and the ThT fluorescence assay to elucidate the recognition determinants between CsgA and CsgC (or CsgH). Guided by the general localisation of NMR perturbation we initially targeted highly-conserved, surface-exposed residues, however we also made simultaneous surface mutations within individual β-strands to alter more dramatically the chemical environment. Mutated CsgCs were checked by 1D NMR to confirm that the surface mutations did not affect overall folding (Fig. S4B).

**Figure 5:**
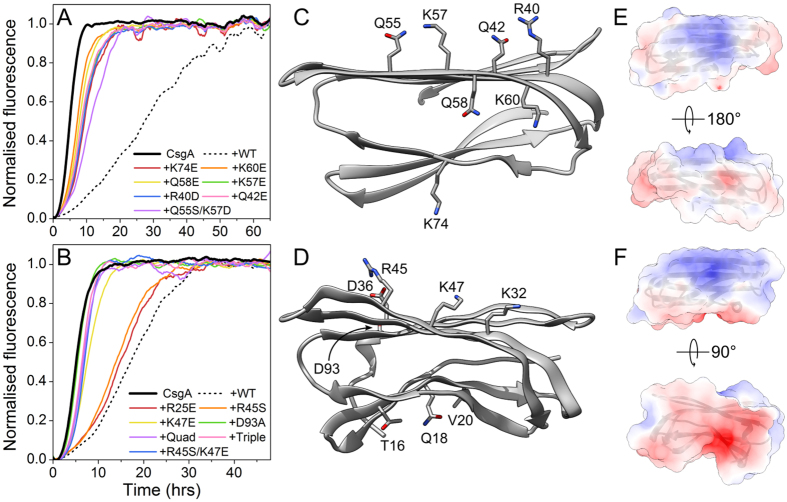
Loss of charged residues in CsgC or CsgH reduces inhibitory potency. Panels (**A,B**) show the relative potency of site-directed mutants of CsgC or CsgH, respectively within the ThT assay. (Define Quad and triple mutants). The raw fluorescence data were smoothed by a Savitsky-Golay filter for clarity. The molar ratio of CsgC/H to CsgA was 1:200. The ‘Triple’ CsgH mutant was T16D/Q18S/V20S. The ‘Quad’ CsgH mutant was K32A/D36S/R45S/K47E. The structural context of each mutation is shown for CsgC and CsgH in panels (**C,D**), respectively. Panels (**E,F**) show the electrostatic surface potential of CsgC and CsgH, respectively. The top sub-panels in each show a conserved positively-charged surface in both proteins.

The most dramatic loss of potency were observed in CsgC mutants in which a positively charged side-chain was switched to a negatively charge one or where negative charge was introduced (see Fig. 5A). We observed a loss of potency for two other charge-related CsgC mutants, whereas point mutations that do not involve charge had no significant effect on CsgC activity (Fig. S4A,B). We also tested the least potent CsgC mutants against FapC amyloid formation in the ThT assay. Once again, interference with charged side-chains had a detrimental effect on inhibition (Fig. S5C). Rather surprisingly, mutation of conserved CXC motif (C37S mutant) or kink-inducing proline residue (P29) within the 2^nd^ β-strand had little effect on potency in this assay (Fig. S5B).

The mutations that caused the severest loss of potency in CsgC are grouped on the protein surface and decorate the perimeter of the β4-β5 edge (Fig. 5C). Two charge mutations that little effect on CsgC inhibition (E30H and D93S) are distance from this edge of the β-sandwich. We probed further for functionally-relevant surface regions by testing CsgC mutants in which 2–3 adjacent, surface-exposed residues along a β-strand were mutated. This shotgun approach revealed that strand 2 and N-terminal half of strand 3 are important to CsgC function whereas strands 1, 6 and 7 and the disordered C-terminus are not (Fig. S6). The common feature associated with every deleterious mutation is that the side-chains are all charged, thus electrostatic interactions appear to be important to the recognition of CsgC by CsgA. The electrostatic surface of CsgC shows that the two β-sheets display a patch of opposite polarity, with the larger β-sheet being mainly positively-charged (Fig. 5E). This general pattern is also conserved in the electrostatic surface of CsgH (Fig. 5F). Guided by our experience with CsgC mutants and our observation of conserved electrostatic potential in CsgH, we constructed a set of mutations in CsgH and tested their potency in the ThT assay against CsgA. Once again, by switching charge polarity at single or multiple sites we observed dramatic loss in potencies (Fig. 5B).

A general conservation of positive charge on the β-sheet surface is seen in many other CsgC and CsgH homologues (Fig. S7). Interestingly we noticed that the closely-related CsgC homologue from *Salmonella typhimurium* (AgfC, 71% sequence identity) displayed a less well-pronounced positively-charged patch, and that three basic residues (each critical for potency within CsgC) were replaced by glutamine within AgfC (R40, K57 and K60). AgfC is unusual in that its pI is only 0.5 units above CsgA/AgfA subunits, whereas for both CsgH and CsgC the pIs are >2 units higher. We therefore hypothesised that AgfC would be less potent inhibitor of *E. coli* CsgA than its cognate inhibitor CsgC. We expressed AgfC in recombinant form using the same construct design as for CsgC and confirmed it was properly folded by NMR and CD spectroscopy (Fig. S8). Despite its high overall sequence similarity, AgfC was significantly worse than CsgC at inhibiting CsgA and FapC amyloid formation, which is in full agreement with our predictions based on electrostatic considerations (Fig. S8). To probe the specificity of the inhibition further, we have tested whether several unrelated protein could inhibit CsgA amyloid formation. Two examples were chosen; *E. coli* maltose binding protein (MBP) which has a low pI but reported periplasmic chaperone activity, and an unrelated highly basic protein DfsB from *Paenibacillus dendritiformis*^24^. MBP had no inhibitory effect on CsgA aggregation, where DfsB did show a modest inhibitory effect, but nowhere near as potent as CsgC/H. Taken together this not only highlights the major influence of positive charge in mechanism of inhibiting CsgA amyloid, but also a role for electrostatic complementarity in CsgC specificity (Fig. S9).

### The inhibitory effect of CsgC is dependent on ionic strength

**Figure 6:**
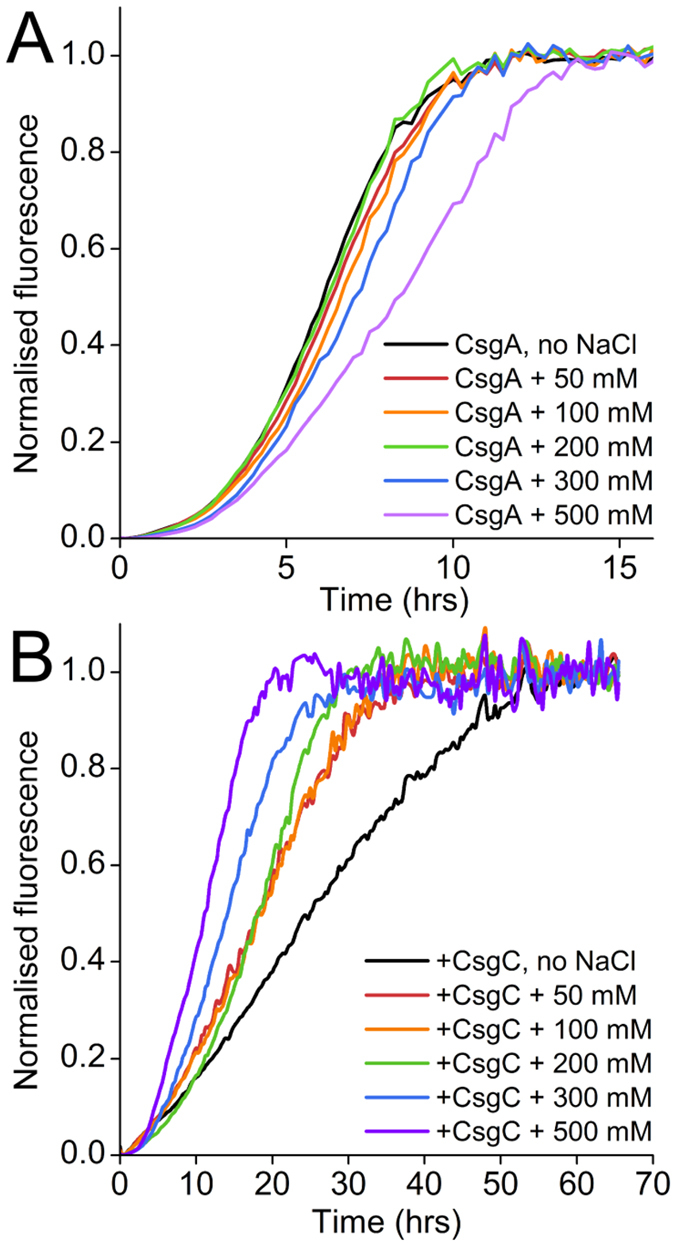
Electrostatic screening effects of NaCl on CsgA amyloid formation and inhibition by CsgC. (**A**) Addition of 0–500 mM NaCl to CsgA samples in the ThT assay causes a minor, dose-dependent reduction in the rate of amyloid formation. The intensity of ThT fluorescence also decreased by up to ~60% across the range Fig. S12. (**B**) The inhibitory potency of CsgC is gradually reduced as the NaCl concentration is increased. CsgC was added at a substoichiometric molar ratio of 1:200 throughout.

We continued to explore the hypothesis that the inhibitory function of CsgC is electrostatically-driven by measuring the potency of CsgC in solutions of increasing ionic strength, which would be predicted to have a negative effect. Addition of NaCl to samples containing CsgA alone resulted in a mild dose-dependent increase in t_½_ (the time when fluorescence = 0.5 × F_max_) and a significant decrease in F_max_ (Figs 6A and S9). The fact that CsgA takes longer to reach the plateau with increasing ionic strength suggests that charge-charge interactions play a role in amyloid formation. Remarkably, we found that the potency of CsgC was also reduced as the NaCl concentration was increased. At 50 and 100 mM NaCl CsgC was slightly less potent, however between 200–500 mM its ability to inhibit CsgA decreased sharply until it had little effect (Fig. 6B). The marked electrostatic screening of CsgC inhibitory potency suggest a key role for electrostatic interactions, likely in driving encounters between CsgC and CsgA.

## Discussion

Almost a decade ago it was reported that *Salmonella* produces different amyloid fibres when its *csgC* gene (*agfC*) is deleted^25^. For many years the role of CsgC with curli biogenesis remained obscure. Its apparent restriction to Enterobacteria, its low concentration in the periplasm, and a lack of a striking deletion phenotype caused it to receive much less attention than other proteins of the curli system.

While CsgC has been shown to be a potent inhibitor of CsgA amyloid formation, the mechanism by which it acts has remained unknown^18^,^23^. The discovery of CsgH, a novel curli-associated protein from α-proteobacteria, and the subsequent solution structure described here reveal that although the amino acid residue sequences of CsgC and CsgH are dissimilar (<20% identity), their structures are remarkably alike. A wider search for CsgC- and CsgH-like proteins revealed at least another distinct clade in *Pseudomonas*. Despite the large differences in sequence to CsgC, *R. palustris* CsgH is also potent inhibitor of amyloid formation by *E. coli* CsgA. This is all the more remarkable when one considers the fact that a single point mutation in CsgC (e.g. K74E) is able to reduce its potency dramatically, and that a much closer functional homologue from *Salmonella* (AgfC) is similarly ineffective.

One of the most striking observations in this work is the central role of charge to the anti-amyloidogenesis mechanisms of both CsgC and CsgH. Electrostatic charge has been shown previously to affect the ability of amyloid subunits themselves to self-polymerise^26^, thus modulating electrostatic encounters may provide a general route to control amyloid. It is worth noting the pI of CsgC/H homologues is on average two pH unit higher that the major curli subunit. Electrostatic attraction would accelerate the encounter between CsgC/H and its substrate CsgA, and potentially allow exposed β-strands (possible the β4-β5 edge) to interact transiently with CsgA and divert it away from any productive amyloid-generating structures.

The high potency of CsgH against the curli amyloid formation and shared electrostatic properties with CsgC suggests a common mode of action. CsgC/H could compete with CsgA monomers for the growing end of amyloid fibril as these are present at only at very low concentrations relative to bulk CsgA. However, the lack of high affinity interaction between any CsgA species and CsgC/H and no evidence of CsgC depletion during amyloid formation suggest that the capping of fibril ends is unlikely, Furthermore, EM observations of CsgA show that the formation of extensive fibrillar networks is delayed in the presence of the inhibitor, which points to CsgC acting primarily at stage prior to the formation of high order fiber species. If CsgC only inhibited elongation then an overall reduction in fibre length should be observed, not solely a delay in their appearance. Consistent with this observation, the global fitting of CsgA amyloid formation kinetics established that CsgC also functions at an early stage, inhibiting the primary nucleation rate with a subsequent smaller effect on elongation (the ratio is approximately 3:2 for the inhibition of nucleation versus elongation). This also rules out a significant depletion of CsgC during the course of the aggregation reaction. We therefore propose that a highly transient interaction occurs with either with an aggregation competent conformation of CsgA or an oligomeric species that represents a key intermediate en route to fibril formation and extension. Our studies also suggest CsgC does not induce any significant degree of secondary structure or multimeric state in CsgA, but increases the conformational distribution of its disordered ensemble.

**Figure 7:**
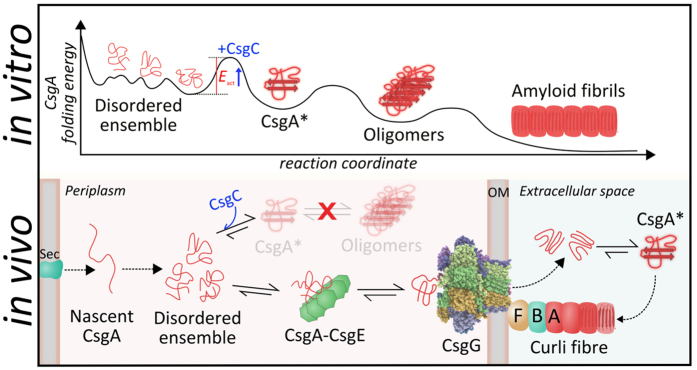
General model that describes inhibition of CsgA by CsgC. The top panel indicates the general folding pathway for CsgA from disordered states via a key nucleation-competent intermediate (CsgA*) that productively multimerises and matures into amyloid fibrils. Although drawn as a monomer, CsgA* may be an oligomer. Fibril ends also capture appropriately structured monomers. Our data are consistent with a model whereby CsgC interacts with disordered monomers or CsgA* to prevent nucleation, effectively increasing the activation energy (*E*_act_) required to form CsgA*. Within the cell (lower panel) this interaction prevents CsgA* formation and keeps CsgA in state readily picked up by CsgE for secretion through CsgG.

We speculate that the precursors of the major nucleating species on the pathway to curli amyloid (CsgA*) manifest themselves as soluble, dynamically-fluctuating, monomeric species, in which the free energy landscape is shallow with a large number of conformations associated with energy minima and small barriers between them (Fig. 7). CsgC-like inhibitors act either by diverting bulk CsgA away from forming CsgA* or by interfering with another species on the nucleation pathway. The lack of the observation of a high-affinity interaction between CsgC and CsgA indicates these are very transient encounters and low-population species. We show that electrostatic attraction drives this encounter which in turn stabilises the disordered ensemble likely through a conformational expansion. This proposed mechanism for CsgC inhibition is akin to that observed for certain types of entropy-driven interactions with disordered proteins. In such examples the presence of the ligand causes enhanced flexibility in the polypeptide, which results in an entropic expansion with an increased number of states becoming accessible^27^. Electrostatic charge has been shown previously to affect the potency of amyloid inhibitors, or the ability of the subunits themselves to self-polymerise^26^,^28^,^29^,^30^. Thus the electrostatic mechanism of amyloid inhibition described here may represent a more general route to controlling amyloid formation.

In the biological context of curli biogenesis, the role of CsgC is likely to maintain CsgA in a secretion-competent conformation within the periplasm (Fig. 7). Recent structures of the curli secretion channel CsgG shows that it is only large enough to capture a monomer of CsgA^31^,^32^,^33^. The conformational restriction imposed by this capture drives secretion of CsgA via an entropy-driven transport mechanism. Clearly, the presentation of CsgA to the CsgG-CsgE secretion complex is likely to be important for its subsequent diffusion through the pore. The ability of CsgC to inhibit the first stages of amyloid formation – nucleation – and maintain a disordered and dynamic ensemble is consistent with its physiological role within the bacteria. Kinetic stabilization of the disordered ensemble would enable the CsgG-CsgE complex to efficiently recruit monomeric CsgA and perform secretion. Given the potency of CsgC against general amyloid formation it is conceivable that organisms may secrete CsgC-like factors to purposely inhibit the formation of amyloid by a competitive species.

## Methods

### Expression & purification of CsgA

The gene encoding the mature form of CsgA (residues 22–151) was amplified by PCR from *E. coli* BL21 (DE3) genomic DNA and ligated into pET28a using *Nco*I/X*ho*I sites. CsgA was expressed and purified as described^34^, with minor modifications as described in Supplementary Material.

### CsgH expression and purification

The *csgH* gene from *Rhodopseudomonas palustris* strain DX-1 was codon-optimised and synthesised by GeneArt (Life Technologies). From sequence/structural comparison with CsgC, we expressed a CsgH construct starting at residue 10. The gene was ligated into pET28 in frame with a C-terminal His tag. CsgH was expressed in *E. coli* SHuffle T7 Express cells (NEB) by growth in LB medium at 37 °C until the OD_600_ reached 0.8 units. Expression was induced at 30 °C by addition of 0.5 mM IPTG. The cells were lysed and clarified by centrifugation at 17,000 rpm for 45 min. Supernatants were purified using Ni-NTA Superflow chromatography (Qiagen). The eluate was gel filtered using a Superdex 75 16/60 size exclusion column (GE Healthcare) equilibrated in 10 mM MES pH 6.5, 0.1 M NaCl.

### CsgC and AgfC expression and purification

CsgC_9-110_ with a C-terminal hexahistidine tag was expressed and purified as described previously^23^. An equivalent construct encoding mature AgfC was also created. The final purification stage of gel filtration was performed in 10 mM Tris-HCl, 100 mM NaCl, pH 8.0.

### FapC expression and purification

FapC was produced as described previously^4^. For a full description of the methodology see Supplementary Materials.

### Structure determination of CsgH

The solution structure of CsgH was solved by standard NMR methods and a combination of manual and automatic assignment. For a full description of the methods used see Supplementary Materials. The coordinates of CsgH were deposited in the Protein Data Bank as accession number 2N59.

### Mutagenesis

Single- and multiple-residue mutants of CsgC, CsgH and CsgA were created using the Q5 Site-Directed Mutagenesis kit (NEB). The expression and purification of site-directed mutants followed the same procedure as wild-type protein. The folding status of each mutant was verified by 1D proton NMR spectroscopy.

### CsgA ThT assay

ThT assays were performed as described previously^18^, using 5 μM CsgA in a buffer containing 50 mM KPO_4_, 5.5 μM ThT, pH 7.4. Inhibitor proteins were added at substoichiometric ratios between 1:100 and 1:1000 from a 5 μM stock. Protein samples (100 μL) were aliquoted into a 96-well plate (Corning, Cat. No. 3881) and sealed with aluminium tape (Costar Cat. No. 6570). Fluorescence measurements at 25 °C were performed using SpectraMax M3e or i3× (Molecular Devices) plate readers with excitation at 438 nm, emission at 495 nm, and a wavelength cut-off at 455 nm. Readings were taken every 15 min and the plate was shaken for 10 sec prior to each reading. Replicate data (n ≥ 3) were averaged and normalised to between 0 and 1.

### FapC ThT assay

Purified FapC was diluted in to 75 μM using ice-cold FapC Assay Buffer. Thioflavin T was added to 40 μM from a 1 mM stock in ethanol. Aliquots of FapC + ThT were transferred to individual microtubes and mixed with varying amounts of inhibitor protein (CsgC, CsgH, AgfC or mutants thereof). A 96-well plate was loaded with 145 μL of sample per well and sealed with foil-backed tape. Fluorescence measurements were obtained every 10 minutes over a 72 hour period under quiescent conditions at 37 °C using a SpectraMax i3× machine. Fluorescence excitation and emission were set to 438 and 480 nm, respectively.

### Kinetic fitting of ThT data

ThT assay data for the addition of CsgC at a range of substoichiometric ratios were fitted simulutaneously to a general kinetic model^22^,^24^. Additions of CsgC 1:400 at timepoints were treated as seeded assays and fitted to a seeded saturated-elongation model to separate relative contributions of nucleation and of elongation to the inhibition.

### Negative Stain Electron Microscopy

CsgA at 35 μM was incubated in Eppendorf tubes at 25 °C with or without CsgC (200:1 molar ratio). Aliquots were taken at 0, 0.5, 1, 2, 3, 5, and 22 hours. For each time point, 10 μL of sample was applied to glow-discharged carbon-coated copper grids and incubated for 2 min before negative staining with 2% uranyl acetate. Samples were imaged at 30,000× on either a Tecnai 12 (FEI) or Tecnai G2 Spirit (FEI) microscope operated at 120 kV. Images were recorded on a 2k TemCam F216 (TVIPS) and an Eagle 2k CCD (FEI) camera (pixel sizes of 4.48 and 7.31 Å/pixel, respectively). For a description of single-particle analyses see Supplementary Materials.

### Biophysical measurements

For details of 1D proton NMR, PFG-NMR, SEC-MALS and ATR-FTIR measurements please refer to Supplementary Materials.

## Additional Information

**How to cite this article**: Taylor, J. D. *et al.* Electrostatically-guided inhibition of Curli amyloid nucleation by the CsgC-like family of chaperones. *Sci. Rep.* **6**, 24656; doi: 10.1038/srep24656 (2016).

## Supplementary Material

Supplementary Information

## Figures and Tables

**Table 1:** Structural restraints and refinement statistics for CsgH.

NMR and Refinement statistics for CsgH	
**NMR Distance and Dihedral Constraints**	
**Unambiguous Distance Constraints**	
Total NOE	1202
Intra-residue	575
Inter-residue	627
Sequential (|i-j|) = 1)	200
Medium range (|i-j|) < 4)	54
Long range (|i-j|) > 5)	373
Ambiguous Distance Constraints	519
**TALOS + Dihedral angle Restraints**	
φ	85
ψ	85
**Structural Statistics**	
**Violations (mean and SD)**	
Distance constraints (Å)	0.25 ± 0.55
Dihedral angle constraints (°)	0.5 ± 0.6
Maximum dihedral angle violation (°)	4.93
**Energies**	
Mean Constraint Violation Energy	283 ± 14.1
Mean amber energy	−2680 ± 43.5
Maximum distance constraint violation (Å)	0.49 ± 0.26
**Mean deviations from idealized geometry**	
Bond length (Å)	0.0062 ± 0.0002
Bond angle (°)	0.629 ± 0.017
**Average Pairwise rmsd (Å) (Residues 1–98)**	
Heavy	1.01 ± 0.10
Backbone	0.42 ± 0.11
**Ramachandran plot**	
% in most favoured regions	94.5% ± 1.3%
% in allowed regions	99.4% ± 0.6%
% in disallowed regions	0%

The statistics shown are for the final ensemble of 20 structures. The error shown represents one standard deviation from the mean.
