# Supplementary material for: Electrostatically-guided inhibition of Curli amyloid nucleation by the CsgC-like family of chaperones
Source: Sci Rep. 2016 Apr 21;6:24656. doi: 10.1038/srep24656 (PMC4838910; doi:10.1038/srep24656)
Supplement: Supplementary Information [file srep24656-s1.pdf]

Supplementary Information

Electrostatically-guided inhibition of Curli amyloid nucleation by the CsgC-like family of chaperones

Jonathan D. Taylor[a], William J. Hawthorne[a], Joanne Lo[a], Alexander Dear[b], Neha Jain[c], Georg Meisl[b], Maria Andreasen[b], Catherine Fletcher[a], Marion Koch[a], Nicholas Darvill[a], Nicola Scull[a], Andrés Escalera-Maurer[a], Lea Sefer[a], Rosemary Wenman[a], Sebastian Lambert[a], Jisoo Jean[a], Yingqi Xu[a], Benjamin Turner[d], Sergei G. Kazarian[d], Matthew R. Chapman[c], Doryen Bubeck[a], Alfonso de Simone[a], Tuomas P. J. Knowles[b], Steve J. Matthews[a,1]

Author Affiliations:

[a]Department of Life Sciences, Imperial College London, London, SW7 2AZ, UK

[b]Department of Chemistry, University of Cambridge, Lensfield Road, Cambridge CB2 1EW, UK

[c]Department of Molecular, Cellular, and Developmental Biology, University of Michigan, Ann Arbor, MI 48109, USA

[d]Department of Chemical Engineering, Imperial College London, London, SW7 2AZ, UK

[1]To whom correspondence should be addressed. Email: s.j.matthews@imperial.ac.uk

Supplementary Methods

Production and purification of CsgA: CsgA22-151-His was expressed in BL21 (DE3) cells grown in Terrific Broth (TB) at 37 °C. Expression was induced at relatively high cell density (OD600 ~1.0) using 0.5 mM IPTG. After 1.5 hrs the cells were harvested by centrifugation in 300 mL aliquots and frozen in liquid N_2. Each aliquot of frozen cells was defrosted and mixed thoroughly in 30 mL Solubilisation Buffer (8 M guanidine hydrochloride, 50 mM potassium phosphate, 0.1 M NaCl, pH 7.8). The sample was sonicated to promote cell lysis and to break up any pre-formed CsgA amyloid seeds. Sonication was achieved using a 4 mm probe tip for 60 seconds in 0.5 sec bursts. After gentle rocking at room temperature for 1-2 hours the lysate was centrifuged at 17,000 rpm to remove insoluble debris. Solubilised CsgA was captured by adding 600 µL TALON resin (50% slurry) and rocking gently for 1 hour at room temperature. The resin was collected by brief centrifugation (3 min, 500 g) and transferred to a 1 mL polypropylene column (Qiagen) and moved to a cold room for subsequent steps. Non-specifically bound protein was washed off the column using sequential aliquots of (a) 1 mL Solubilisation Buffer; (b) 1 mL ice-cold 1.6 M guanidine hydrochloride, 50 mM potassium phosphate, 0.1 M NaCl, pH 7.8; (c) 1 mL ice-cold 50 mM potassium phosphate, 0.1 M NaCl, pH 7.8; (d) 2 mL ice-cold 50 mM potassium phosphate, 0.1 M NaCl, 2 mM imidazole, pH 7.8. CsgA was eluted directly into a 4 mL Amicon centrifugal concentrator (30,000 MWCO, pre-washed in Assay Buffer) using 1.6 mL ice-cold 50 mM potassium phosphate, 0.1 M NaCl, 500 mM imidazole, pH 7.8. Resin washing and elution steps were performed in under 5 minutes to minimize pre-assay fibrillation. The sample was centrifuged for 10 minutes at 4000 g at 4 °C to remove aggregates. Finally, the filtrate was injected through a 5 mL HiTrap Desalting column (GE Healthcare) pre-equilibrated in ice-cold Assay Buffer (50 mM potassium phosphate, pH 7.4). Protein concentration was measured using a Nanodrop spectrophotometer. A typical purification yielded ~2 mL 25 uM CsgA. Where additional protein sample was required the aliquots were kept separate throughout the purification but combined before desalting into assay buffer.

FapC expression and purification: BL21 (DE3) *E. coli* cells were transformed with the pET28a vector harbouring the gene for FapC (residues 25-250) from the *Pseudomonas fluorescens* strain UK4 without the signal sequence (residues 1-24) and with 6 histidine residues in the C-terminus. Cells were grown on LB-agar plates with kanamycin at 37 °C. Colonies were transferred to LB medium with kanamycin and grown to OD600 ~1. Protein expression was induced by adding IPTG to a final concentration of 1 mM followed by 3 h incubation. Cells were harvested and resuspended in 20 mL/L culture 50 mM Tris-HCl pH 8, 8 M guanidinium hydrochloride and lysed by sonication. Cell debris was removed by centrifugation for 30 min at 5000 g. The supernatant was loaded onto a His-trap column (Super Nickel NTA resin, Generon). The column was washed with increasing concentrations of imidazole (0, 30, 60 and 120 mM in 50 mM Tris-HCl pH 8, 8 M guanidinium chloride) and eluted with 9 mL (2 CV) 300 mM imidazole, 50 mM Tris-HCl pH 8, 8 M guanidinium chloride and flash-frozen in liquid N_2. The fractions were analyzed by SDS-PAGE following ethanol precipitation. Elution fractions containing pure FapC were pooled and concentrated down to 2.5 mL using a pre-washed Vivaspin 20 (5kDa MWCO) device and loaded onto a PD-10 desalting column pre-equilibrated in 20 mM sodium phosphate pH 7.2 (FapC Assay Buffer). The protein was eluted with 3.5 mL of FapC Assay Buffer, filtered through a pre-washed 0.22 µM filter, and stored on ice. The concentration of FapC was measured using a spectrophotometer (MW = 25.0 kDa, ε_{280} = 10095 $M^{-1}cm^{-1}$).

Structure determination of CsgH. Despite extensive crystallisation trials CsgH failed to produce diffraction-quality crystals and instead we solved its structure using standard NMR methods. ^{13}C,^{15}N-labelled CsgH was produced by expressing the protein in M9 minimal

medium supplemented with U-^{13}C-Glucose and ^{15}N-Ammonium chloride and overnight induction at 25 °C by addition of IPTG to 0.5 mM. The protein was purified as described above and concentrated to 400 µM. D_2O was added to a concentration of 10% [v/v]. D_2O was added to 10% for all experiments apart from the ^{13}C NOESY-HSQC and H-H NOESY experiments which were carried out in 100% D_2O. All NMR spectra were acquired at 292 K on Bruker Avance-III HD 950, Avance-II 800 and Avance-III 600 spectrometers. Triple-resonance HNCACB, CBCA, HNCO and HN(CA)CO spectra were used together with a ^{1}H-^{15}N HSQC spectrum to obtain the backbone assignments. Side-chain chemical shift assignments were obtained using HBHA(CO)NH, CC(CO)NH and HCCH-TOCSY, ^{1}H-^{13}C HSQC and H-H NOESY spectra. Structural distance constraints were obtained from ^{15}N NOESY-HSQC, ^{13}C NOESY-HSQC and H-H NOESY spectra (each with a mixing time of 100 ms). Spectra were processed and assignments made as described previously [1]. Dihedral angles were obtained from the chemical shift assignments using TALOS + [2], the resulting predictions were used for the structure calculation. NOE peaks were picked manually in CcpNmr 2.4.0 without any manual assignments. Automatic NOE assignment and structure calculation was carried out using ARIA 2.3/CNS 1.1 software packages [3]. An additional constraint was included to direct the formation of a disulphide bond between Cys4 and Cys90 in the protein structure. A set of 300 structures were calculated in the final iteration and the 30 best structures were refined in water, from which the 20 lowest-energy structures. The coordinates of CsgH were deposited in the Protein Data Bank (www.rcsb.org) under PDB-ID accession number **2N59**.

Circular dichroism (CD): Protein samples were diluted in 50 mM KPO_4 pH 7.4 to ~0.15 mg/mL and placed in a 1 mm pathlength cuvette (Hellma Analytics, 100-QS). Triplicate CD spectra were recorded at 25 °C using a Chirascan Spectrometer (Applied Photophysics) across the wavelength range 190-260 nm in 0.5 nm steps. The resultant data were averaged, buffer-subtracted, and smoothed with a 17-point Savitsky-Golay digital filter for clarity.

^{1}H-NMR: Protein samples (0.5 mL) were placed in a clean NMR tube and supplemented with 5-10 % [v/v] 100% D_2O. One-dimensional 1H-NMR spectra were recorded on a Bruker Avance-III 600 spectrometer, with 128-512 scans dependent on sample concentration. Spectra were processed within TopSpin 3.2 (Bruker).

PFG-NMR: A 0.5 mL sample of 20 µM freshly-purified CsgA (+5% [v/v] D_2O) was placed in clean NMR tube. A series of PFG-edited spectra were recorded (128 scans each) with a range of gradient field strengths using a Bruker Avance-III 600 spectrometer. Spectra were processed identically within TopSpin (Bruker) and the integral of the methyl peak from each (between 0.5 and 1.0 ppm) was calculated and normalised to between 0 and 1, with the latter value corresponding to a pulse sequence of zero field gradient. The self-diffusion coefficient was calculated within Excel using in-house scripts.

SEC-MALS: A Superdex 200 10/300 analytical gel filtration column (GE Healthcare) was equilibrated in 50 mM KPO_4, 50 mM NaCl pH 7.4 buffer overnight. Samples of CsgA alone (0.3 mg/mL) or pre-mixed with CsgC (200:1 molar ratio) were injected onto the column. Inline static light scattering (3-angles) and differential refractive index data were collected using miniDAWN TREOS and Optilab T-rEX devices (Wyatt Technologies). Molecular weight calculations were performed in ASTRA 6 with the dn/dc value of CsgA set to 0.185 mL/g.

ATR-FTIR: The infrared spectra were recorded using a Bruker IFS 66/S FTIR spectrometer (Bruker optics) equipped with a multi-reflection ZnSe ATR accessory (Gateway™ ATR Accessory, Specac limited).The spectrometer and the ATR accessory were continuously purged with dry air. A 1 mL solution of CsgA with and without the presence of CsgC at a

concentration of 40 µM was pipetted onto the ATR crystal. A spectrum was measured immediately and then again 24 hours later. For each spectrum an average of 128 scans was taken. The spectra were recorded at room temperature using a spectral resolution of 4 cm-1. A clean ZnSe crystal was used for the background scan. All data analysis was carried out in matlab. The spectral contribution of water was subtracted from the protein spectrum using the method detailed in Powel et al. The spectra were then baseline corrected and normalised for equal area between 1600 cm^{-1} and 1700 cm^{-1}.

Negative stain EM single particle analysis:. 56 short segments along CsgA double filaments were extracted from the 22 hour time-point and analysed by reference-free alignment and classification with Xmipp [4]. From left to right in Figure 3 the four class averages contain 9, 8, 14 and 20 images, respectively.

Kinetic analysis of CsgA aggregation in isolation. The kinetics of amyloid fibril formation can be described by general kinetic models, where fibril mass and numbers of fibrils are considered explicitly as functions of time. Bulk unseeded aggregation experiments of CsgA were performed, at a range of different CsgA concentrations, and under the same conditions as used in the CsgA+CsgC aggregation experiments (see Methods). Aggregate mass was measured by ThT fluorescence, and the resulting normalised ThT fluorescence data was analysed using the AmyloFit online fitting tool[5]. Good fits could only be obtained to a model featuring just 2 microscopic processes: Formation of aggregates by nucleation of new fibrils (rate constant k_n, reaction order n_c), and aggregate growth through monomer addition to the ends of existing fibrils (rate constant k_+) (Fig.S10). We are able to say with confidence that secondary processes, such as fragmentation and secondary nucleation, do not occur under these conditions. The moment equations, for number and mass concentration of fibrils, can then be written as:

$$\frac{dP}{dt} = k_n m(t)^{n_c} \tag{1}$$

$$\frac{dM}{dt} = 2k_+ m(t) P(t) \tag{2}$$

where P is the number concentration of fibrils, and M is their mass concentration. These can then be solved in closed form[5], giving the following solution for the mass concentration:

$$\frac{M}{m_{\mathrm{tot}}} = 1 - \frac{m_0}{m_{\mathrm{tot}}} \left(\frac{1}{\mu} \cosh\left(\sqrt{\frac{n_c}{2}} \mu\lambda t + \nu\right) \right)^{-\frac{2}{n_c}} \tag{3}$$

where the definitions of the parameters are

$$\lambda = \sqrt{2k_+ k_n m_0^{n_c}}$$

$$\alpha = \sqrt{\frac{k_+ n_c}{k_n m_0^{n_c}} P_0}$$

$$\nu = \log(\alpha + \mu)$$

$\mu = \sqrt{1 + \alpha^2}$

When non-seeded initial conditions, $P(0) = M(0) = 0$, are used, this reduces to the simpler form:

$$\frac{M}{m_{\mathrm{tot}}} = 1 - \left(\cosh(\sqrt{\frac{n_c}{2}}\lambda t)\right)^{-\frac{2}{n_c}} \qquad (4)$$

We note that this has a different mathematical form to the expressions for mass concentration in models that include secondary processes. Qualitatively it is not of sigmoidal form, and does not therefore show a classical lag phase.

Eq.(4) allows the constants n_c and k_c (= k_+k_n) to be determined from the time evolution of the mass concentrations of fibrils for any initial monomer concentration in the absence of seeding. We obtained k_c=7850$M^{-1}h^{-2}$, and n_c=1

Kinetic analysis of CsgC inhibition of CsgA aggregation. We then turned our attention to the effect of CsgC at sub-stoichiometric concentrations on the kinetics of CsgA aggregation. An experiment was performed in which CsgC at different concentrations was added to CsgA before starting aggregation, and ThT fluoresence was measured over time. This experiment showed lower plateau values of ThT fluorescence with increasing CsgC. It was shown through NMR spectroscopy (Fig S11) that this was due to a reduction of fluorescence rather than a reduction of total aggregate mass; therefore, it was appropriate to normalize all the reaction profiles to the same plateau value.

We tested the normalized data against the same kinetic model used in CsgA-only fitting and it was found that in general, good fits can be obtained to these equations for both inhibited and uninhibited experimental data, for reaction order n_c=1; however different values for the rate constant k_c are obtained for different inhibitor concentrations. We see a clear trend of k_c decreasing steadily with increasing CsgC concentration (see Fig.4A).

In order to determine the relative inhibition of nucleation and of elongation, it was necessary to use other experiments. This is not straightforward as bulk aggregation experiments are somewhat sensitive to small variations in experimental conditions. It is therefore preferable to be able to determine the relative inhibition from one experiment or set of experiments. For this purpose, an experiment was designed and carried out in which a number of samples of CsgA are allowed to start aggregating simultaneously, and then CsgC (at a stoichiometry of 1:400) was added at various later time points to different samples. From the shape of the aggregation curves it was clear that the fluorescence efficiency of ThT is only affected for ThT binding to aggregate mass formed after addition, so we re-normalized only the parts of the aggregation curves occurring after addition. The data is truncated at the point of CsgC addition, and can then be interpreted as a seeded experiment, with the seed concentration equal to the aggregate concentration at the time of addition.

Given that n_c=1, we can write the effective seed concentration $P(0; \tau)$ as a function of k_n^0, k_c^0 and τ, where k_n^0 is the uninhibited rate of primary nucleation, and τ is the time of addition of inhibitor:

$$P(0;\tau) = \frac{k_n^0 m_{\mathrm{tot}}}{\sqrt{k_c^0 m_{\mathrm{tot}}}} \tanh(\sqrt{k_c^0 m_{\mathrm{tot}}}\tau) \qquad (5)$$

This allows us to simplify the parameter α from equation (3) to:

$$\alpha(\tau) = \sqrt{\frac{k_c}{k_c^0}\frac{k_n^0}{k_n}} \tanh(\sqrt{k_c^0 m_{\mathrm{tot}}}\tau) \qquad (6)$$

$k_n{}^0$ is unknown, as are k_n and k_c (= k_+k_n); however, we can see from the functional form of Eq.(3) and Eq.(6) that the aggregate mass depends only on the combined quantities k_+k_n and $k_n{}^0/k_n$ and the correct value of $k_n{}^0$ is hence not needed in order to determine the inhibition of nucleation. By fitting the truncated data to Eq.(6), and taking the value for $k_c{}^0$ from the fitting to the uninhibited CsgA data, we obtained values for k_+k_n and $k_n{}^0/k_n$ of ~2000 and ~2.5 respectively. Thus we immediately see that nucleation is inhibited by a factor of 3. Combining parameters further allowed us to determine the factor by which elongation is inhibited to be ~1.5. Thus we can conclude that CsgC inhibits both nucleation and elongation of CsgA in this parameter regime, and our experiments indicate that they are inhibited in an approximate ratio of 5:3 respectively.

1 Marchant, J., Sawmynaden, K., Saouros, S., Simpson, P. & Matthews, S. Complete resonance assignment of the first and second apple domains of MIC4 from Toxoplasma gondii, using a new NMRView-based assignment aid. *Biomol. NMR Ass.* **2**, 119-121, (2008).

2 Shen, Y., Delaglio, F., Cornilescu, G. & Bax, A. TALOS+: a hybrid method for predicting protein backbone torsion angles from NMR chemical shifts. *J. Biomol. NMR* **44**, 213-223, (2009).

3 Bardiaux, B., Malliavin, T. & Nilges, M. ARIA for solution and solid-state NMR. *Methods Mol. Biol.* **831**, 453-483, (2012).

4 de la Rosa-Trevin, J. M. *et al.* Xmipp 3.0: an improved software suite for image processing in electron microscopy. *J. Struct. Biol.* **184**, 321-328, (2013).

5 Meisl, G. *et al.* Molecular mechanisms of protein aggregation from global fitting of kinetic models. *Nat. Protoc.* **11**, 252-272, (2016).

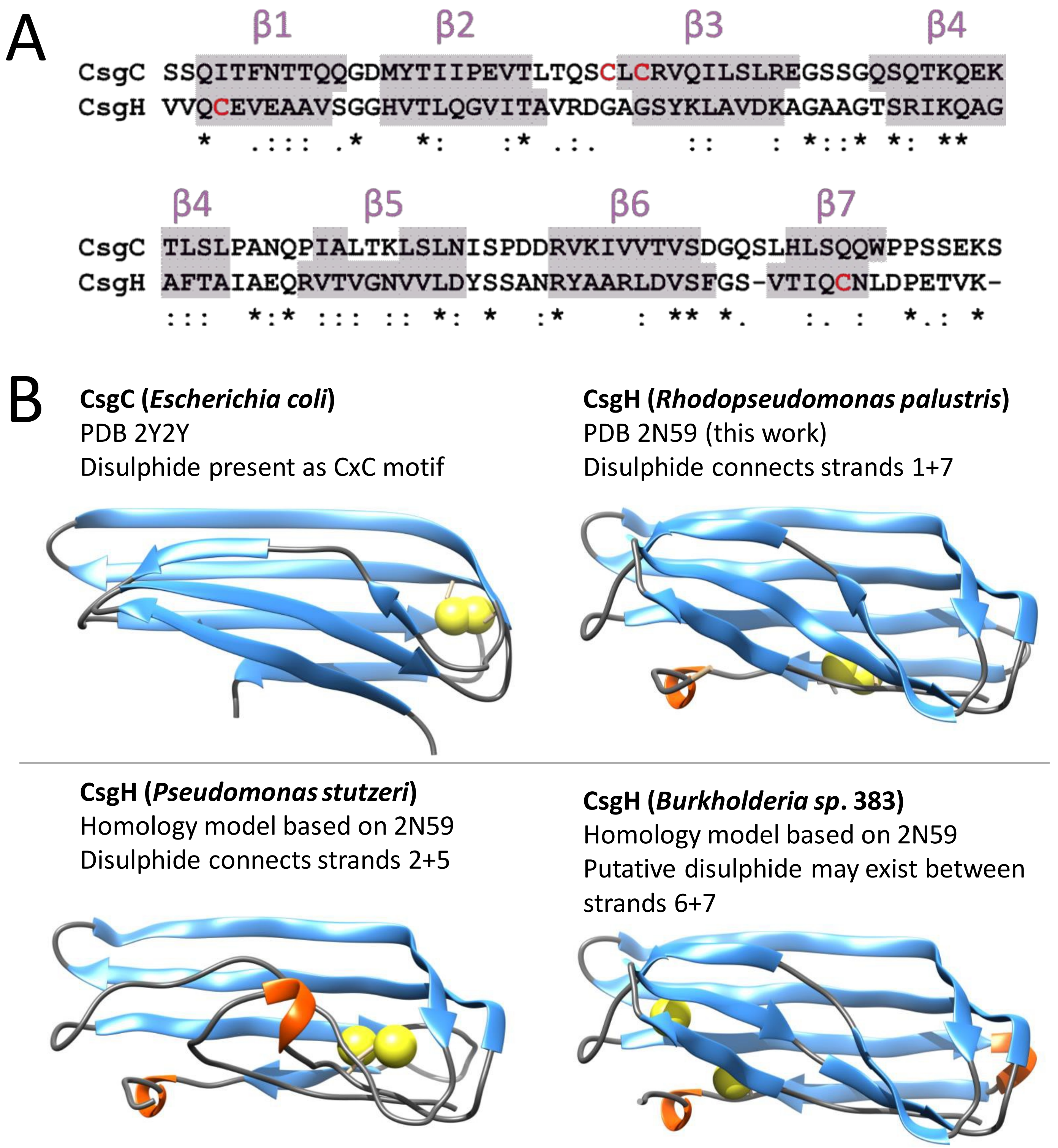

Figure S1. Sequence/structural comparisons within the CsgC and CsgH familes. (A) Sequence alignment of *E. coli* CsgC with CsgH from the α-proteobacterium *Rhodopseudomonas palustris*. The predicted β-strands (highlighted in purple) in CsgH match closely those observed in CsgC. **(B)** Alternate disulphide bonding patterns in CsgH vs CsgC. The majority of CsgH homologues possess a disulphide connecting the first and last stands, as shown in our structure of CsgH (top right). Sulphur atoms are shown as yellow spheres. At least two other connecting disulphides also occur, for example in *Pseudomonas* and *Burkholderi*a (lower panels). Phyre[2] was used to create homology models for these CsgH sequences on the basis of our CsgH structure. The known structure of CsgC (PDB code 2y2y) is shown for comparison (top left).

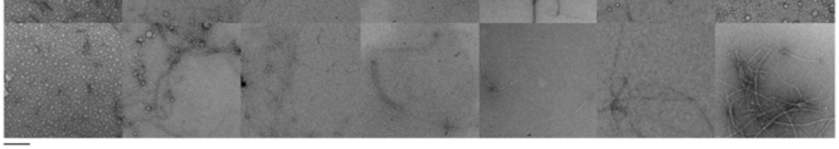

A

B

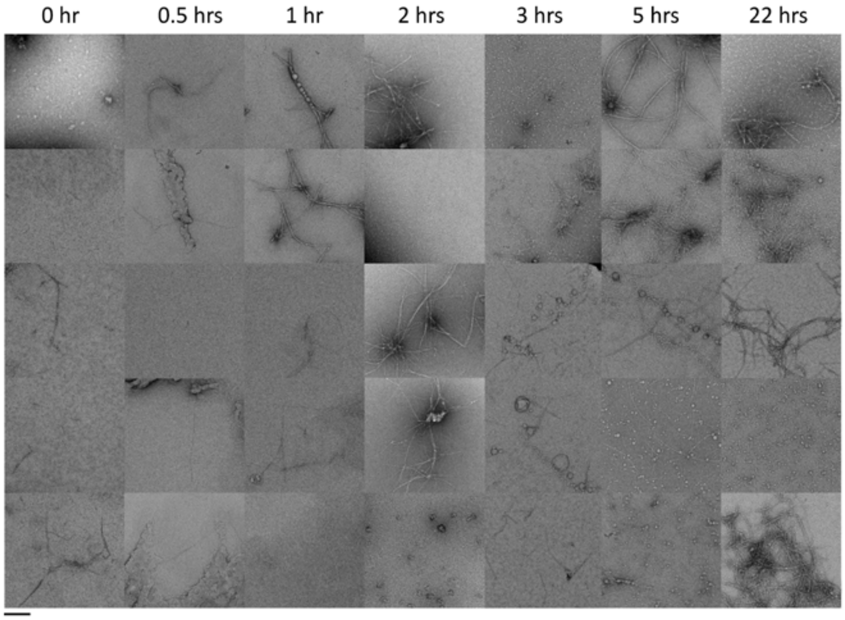

Figure S2. Negative-stain EM analysis of aggregating CsgA. (A) CsgA (35 µM) was purified and allowed to form fibres in a 1.5 ml microtube at room temperature without agitation over 24 hours. Small aliquots were periodically removed and used to make EM grids. Each grid was carefully analysed and a representative set of images is displayed for each time-point shown. Scale bar, 200 nm. **(B)** Negative-stain EM analysis of aggregating CsgA in the presence of CsgC (200:1 CsgA:CsgC) under identical conditions as in (A)

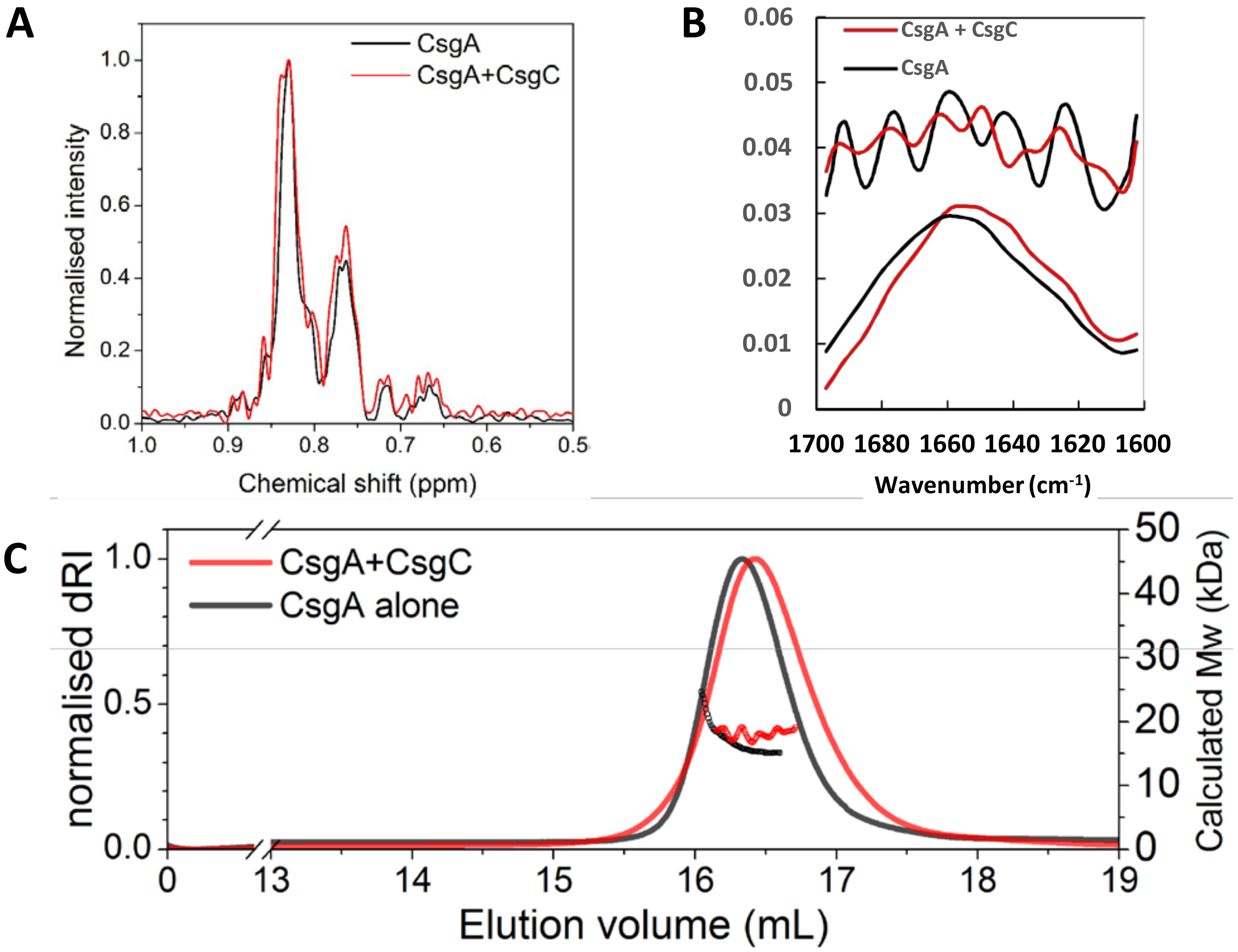

Figure S3. CsgC influences the average ensemble structure of CsgA. (A) ^{1}H-NMR spectra of 5 µM fresh CsgA in the absence (black) or presence (red) of CsgC (molar ratio 200:1). Only the methyl region is shown for clarity. The spectrum observed for the mixture suggests that CsgC directs CsgA into a different average structure. **(B)** (bottom) ATR-FTIR spectrum of freshly purified CsgA. Spectra of 40 µM CsgA in the absence or presence of CsgC (200:1 molar ratio). (top) Second derivative of the ATR-FTIR spectrum. The ATR-FTIR spectra for the 24 hrs time points are shown in main Figure 3D for comparison. **(C)** SEC-MALS profiles of freshly purified CsgA alone *vs.* CsgA + CsgC (molar ratio 200:1). The calculated population weight-averaged molar mass for CsgA in the presence of CsgC (red diamonds, ~16.5 kDa) is fairly stable across the peak implying monodispersity. Conversely, CsgA alone (black diamonds) displays slight polydispersity consistent with formation of small oligomers by a fraction of the overall CsgA population. Samples in these solution experiments (**A-C**) are freshly prepared and data collected within minutes. There is no detectable evidence in any of our assays for large oligomers at this stage, so the monomer/small oligomer concentration represents close to 100% of the sample.

A

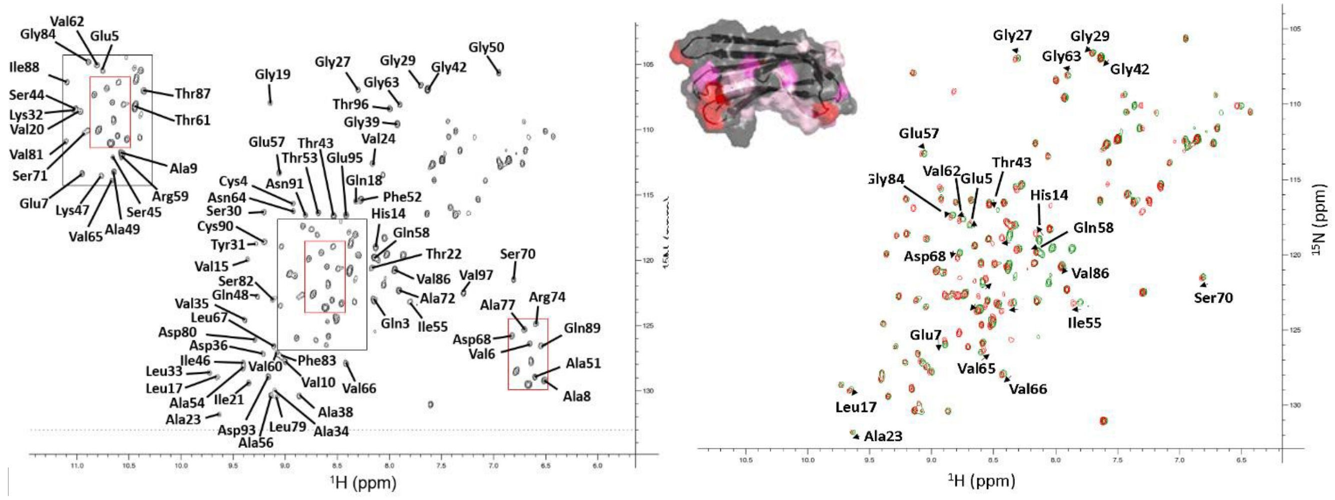

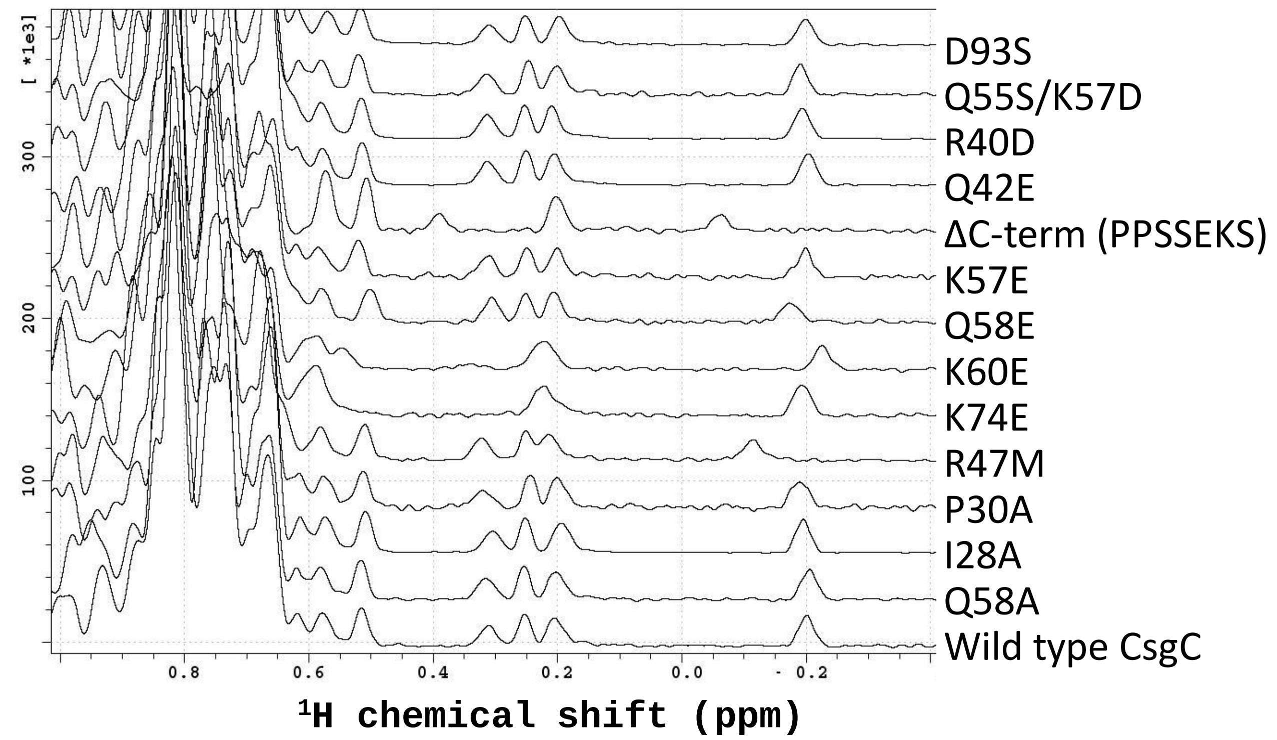

Figure S4. NMR analysis of CsgH-CsgA interaction and CsgC site-directed mutants. (A) Assigned ^{1}H-^{15}N HSQC spectrum of CsgH used for the solution structure dtermination (left) and an overlay (right) of spectra recorded on 20 µM CsgH in the absence (free) and presence (red) of 3-fold excess of CsgA. A surface representation showing the affected resonances is shown as an inset in the same orientation as the Figure 5 displaying the key charge mutation. Similar affects were also see in the NMR spectra of CsgC in the presence of CsgA **(B)** To confirm folding, CsgC and CsgH mutants were subject to NMR spectroscopy. Methyl groups in the vicinity of aromatic rings (i.e. within the stable hydrophobic core of a protein) tend to display distinct chemical shifts below 0.5 ppm. It should also be noted that upfield-shifted methyl group are highly sensitive to very small changes in local structure. The distinct methyl patterns for some mutation that do not remove methyl groups (e.g. K60E and K74E) is due to small local changes in structure. The presence of high field methyl groups in all spectra of CsgC/CsgH mutants, confirming that the altered residue has not affected the overall fold of the protein.

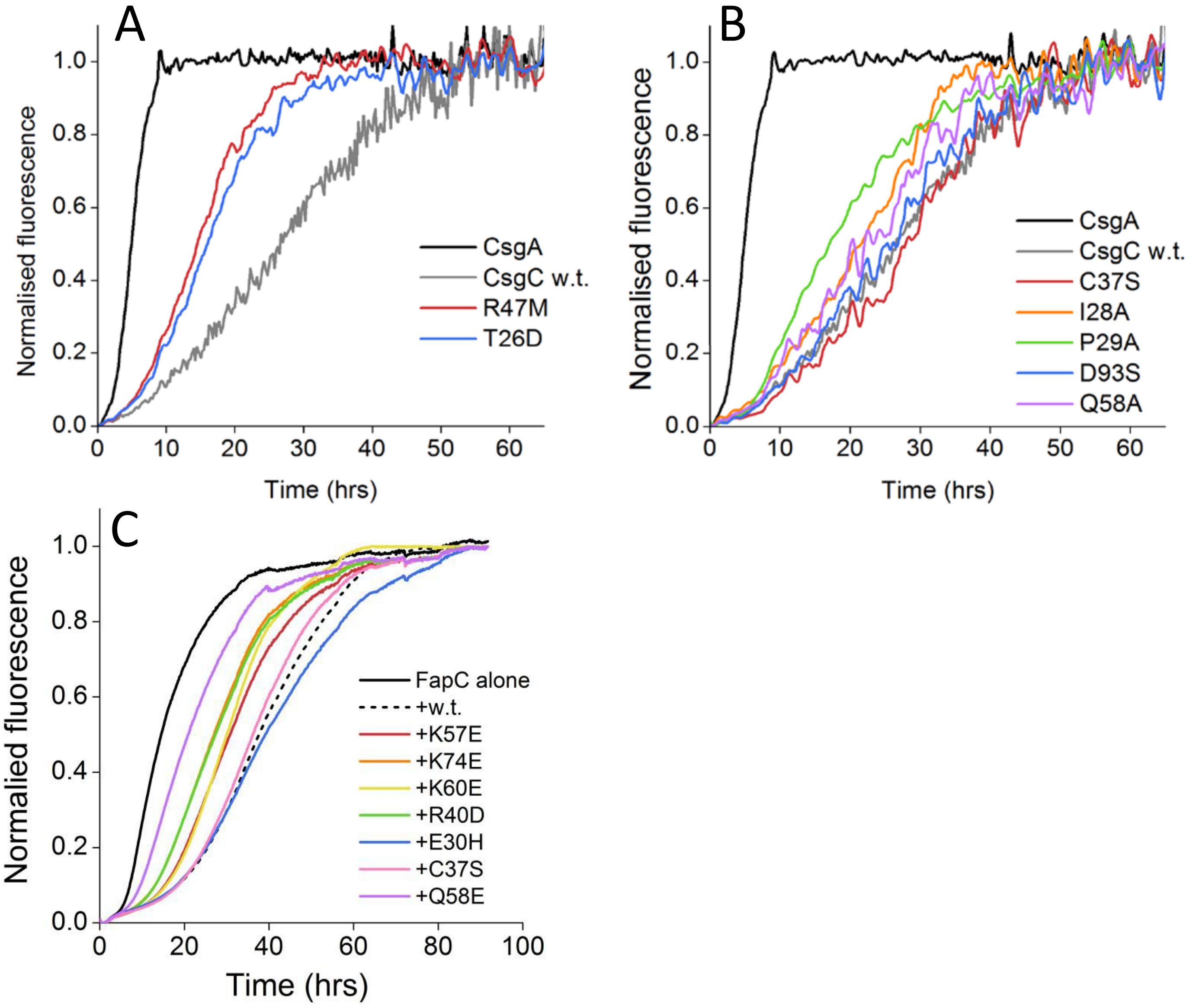

Figure S5. Additional ThT assay data for CsgC mutants. (**A**) CsgC mutants with slight loss of potency. (**B**) CsgC mutants with similar potency to wild type protein. (**C**) CsgC mutants tested against FapC amyloid formation. Residue numbering for CsgC is for full length protein. (i.e. inclusive of signal sequence).

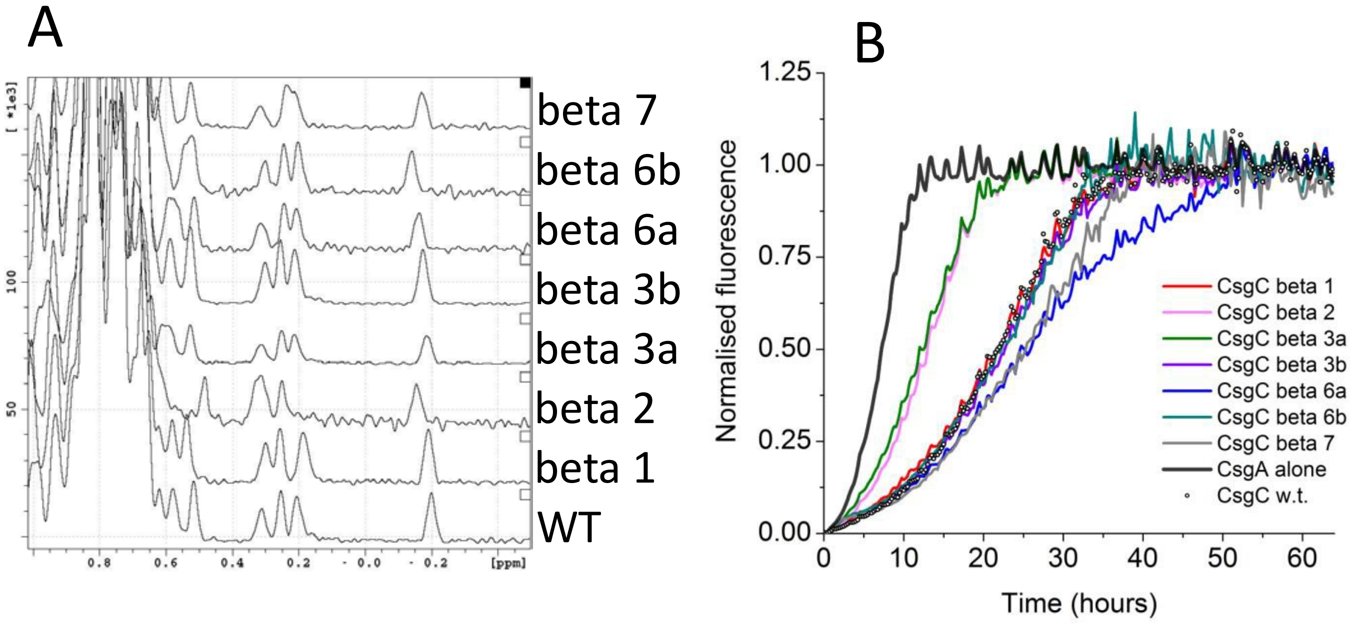

C

Construct	Mutations	Potency
CsgC beta 1	T15K / N17S / T19D	Equivalent to w.t.
**CsgC beta 2**	**T26D / I28S / E30H**	**Less than w.t.**
**CsgC beta 3**	**R40S / Q42S / L44E**	**Less than w.t.**
CsgC beta 3b	L44E / L46H / E44S	Equivalent to w.t.
CsgC beta 6a	R84S / K86E / V88S	Slightly more than w.t.
CsgC beta 6b	V88S / T90E / S92G	Equivalent to w.t.
CsgC beta 7	S100K / Q102G	Slightly more than w.t.

Figure S6. ThT assay data for CsgC multiple mutants. Simultaneous mutation of 2-3 contiguous, surface-exposed side-chains within individual strands of CsgC were created. (**A**) ^{1}H NMR was used to confirm native folding. (**B**) Each mutant was tested for potency in the ThT assay against CsgA. (**C**) The table shows the phenotype of each mutation. Mutation of strand 2 or the N-terminal half of strand 3 leads to a significant loss in potency. Residue numbering refers to the full length CsgC sequence.

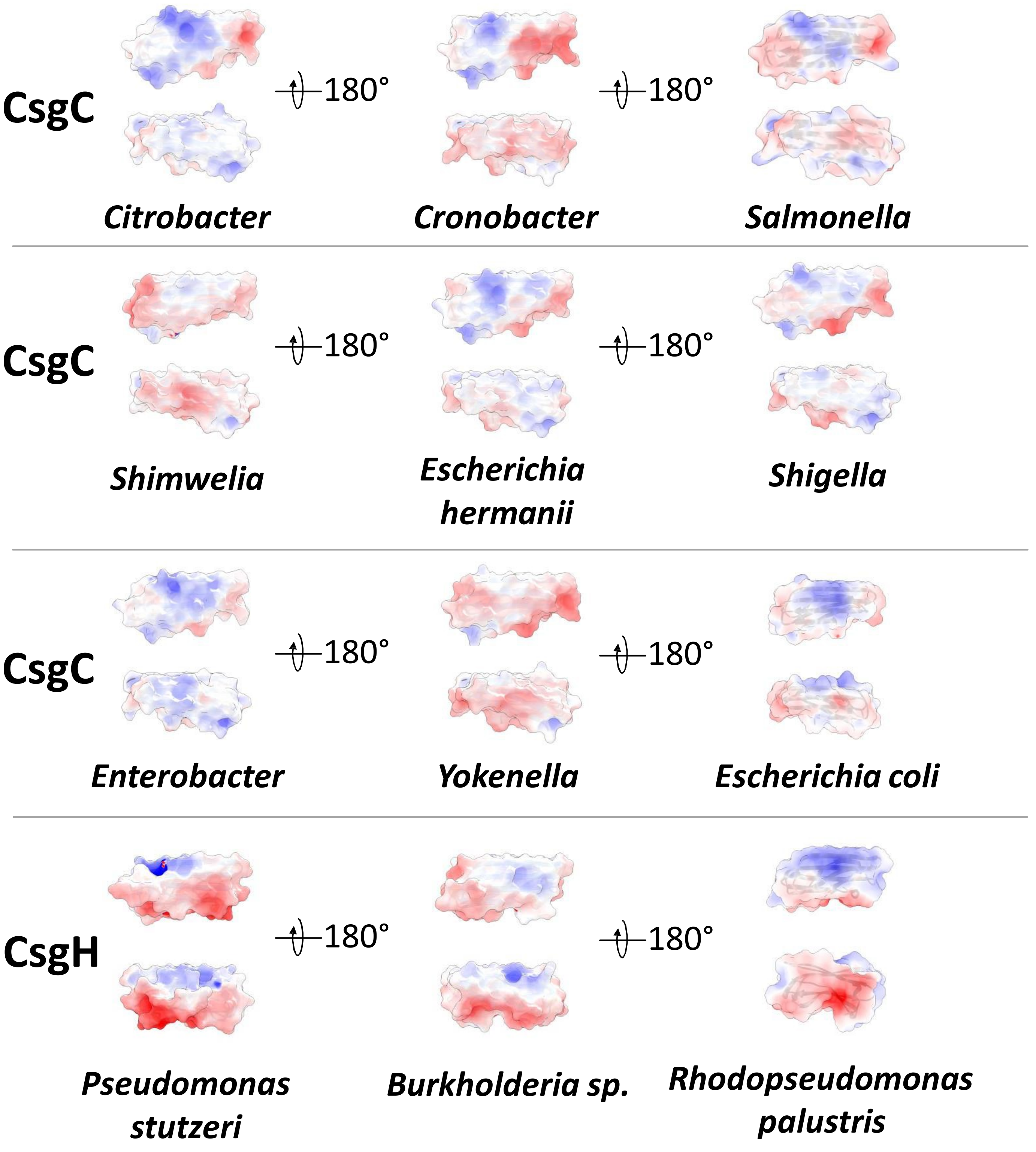

Figure S7. Conservation of electrostatic surfaces across CsgC/CsgH homologues. The crystal structure of *E. coli* CsgC was used to create homology models of CsgC from Enterobacteria. Similarly, the NMR CsgH structure from R. palustris was used to build homology models of two representative CsgH-like proteins from *Pseudomonas* and *Burkholderia*. Electrostatic surfaces were calculated using PDB2PQR server (http://nbcr-222.ucsd.edu/pdb2pqr_2.0.0) and visualised in Chimera.

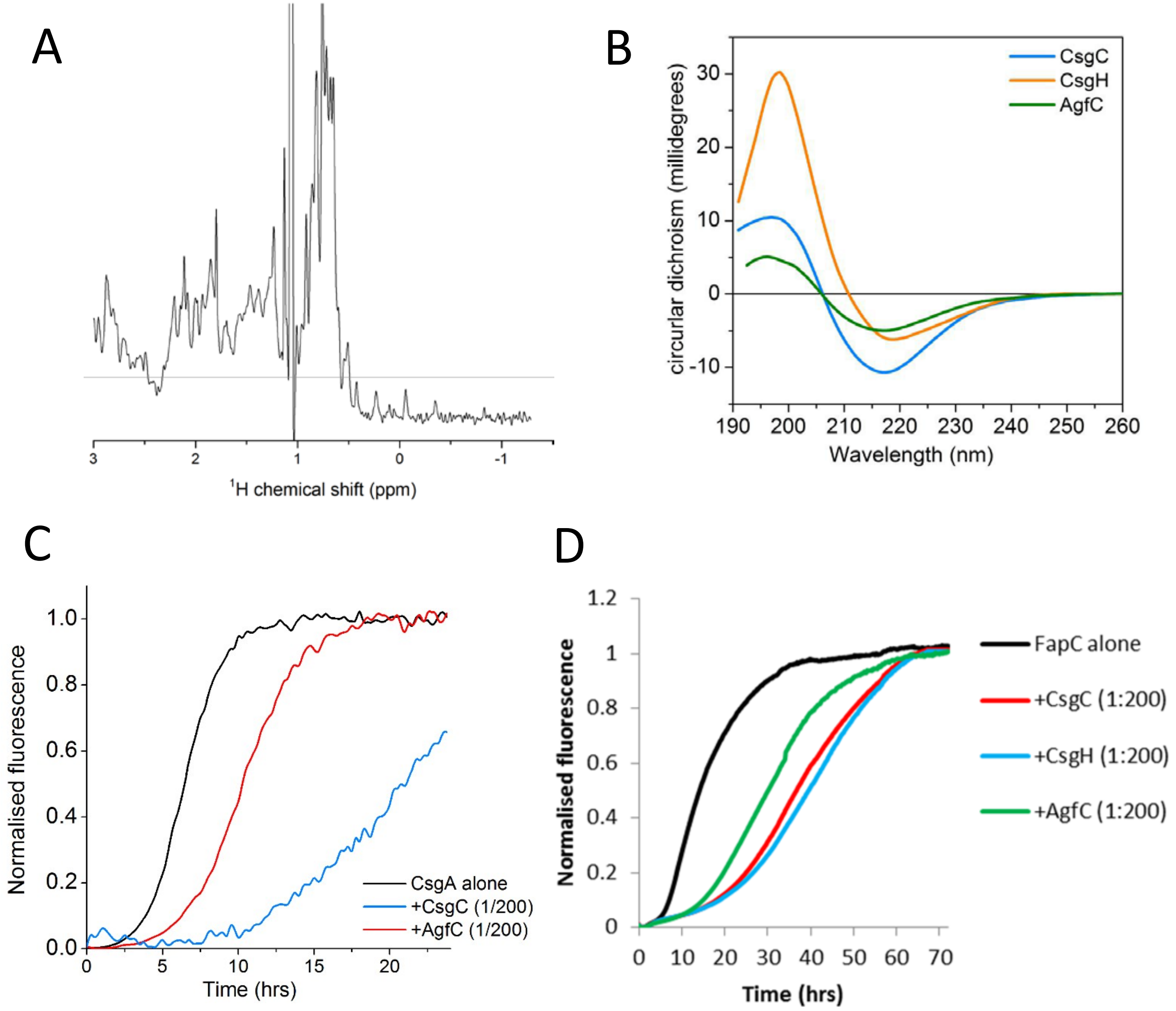

Figure S8. Structure and functional data on AgfC. (A) ^{1}H-NMR spectrum of AgfC, aliphatic region only. The ring-current shifted methyl peaks below 0 ppm and favourable spread of proton resonances shows that AgfC forms a stable fold with minimal disordered or unfolded regions. (**B**) Circular dichroism spectroscopy of CsgC, CsgH and AgfC. Each spectrum shows the characteristic minima and maxima for β-rich protein. (**C**) Comparing the potency of AgfC with CsgC. The inhibitor proteins were added to CsgA in a substoichiometric ratio of 1:200 and the formation of amyloid was monitored by ThT fluorescence. (**D**) Comparison of the inhibitory potency of CsgC, CsgH and AgfC in restricting the amyloid formation of FapC (1:200 molar ratio).

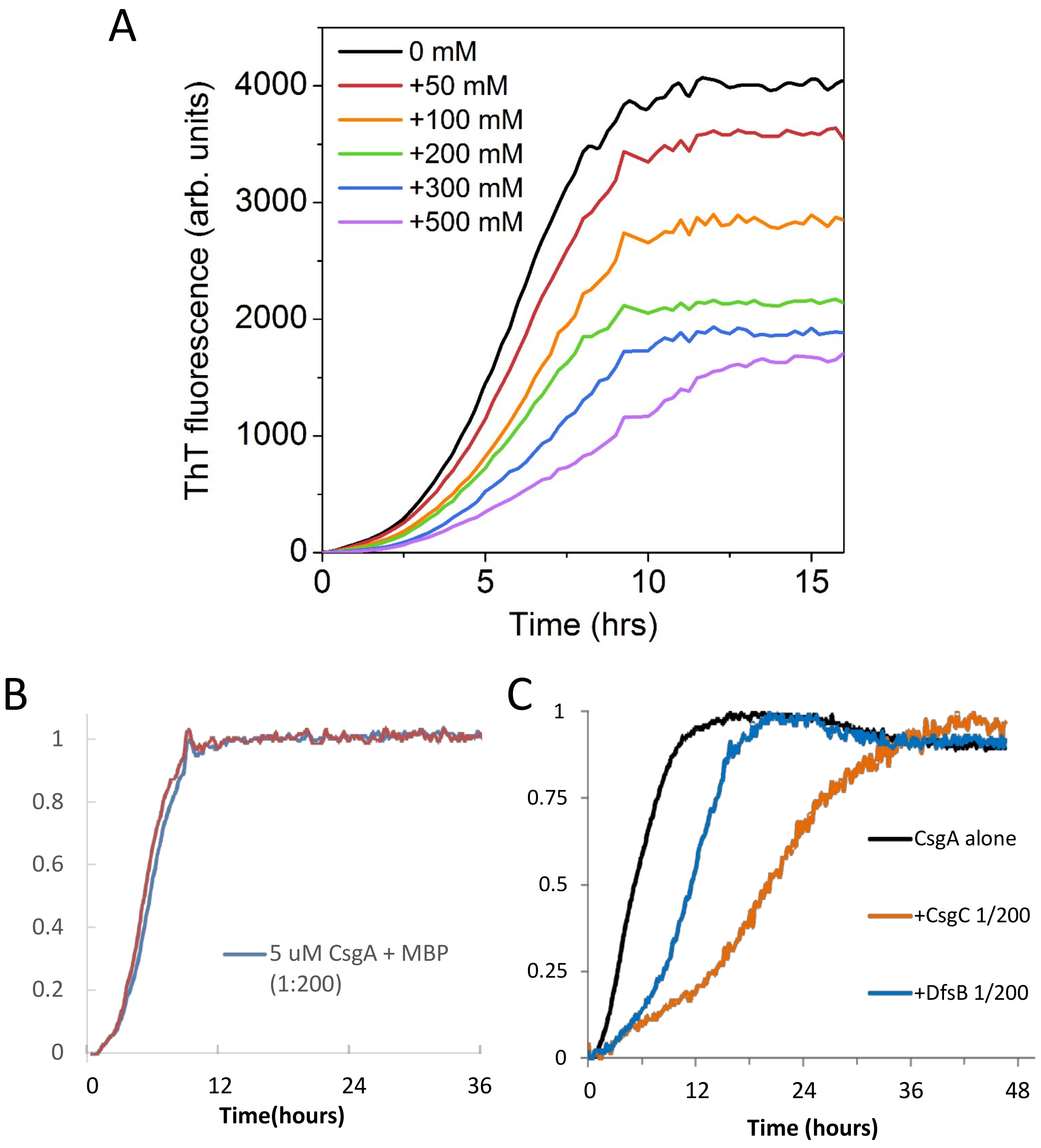

Figure S9. Effect of NaCl on CsgA amyloid formation. (A) Addition of NaCl from leads to a non-linear decrease in maximal fluorescence in the plateau region. NaCl clearly causes slight changes in the ultrastructure of the amyloid fibre, which affects ThT binding conformation. Addition of NaCl also causes a gradual reduction in the rate of fibre formation. The data in this figure were normalised to between 0 and 1 and shown in Fig. 6A. **(B)** Comparing the potency of *E. coli* Maltose binding protein with CsgC. The inhibitor proteins were added to CsgA in a substoichiometric ratio of 1:200 and the formation of amyloid was monitored by ThT fluorescence. **(C)** Comparing the potency of highly basic protein DfsB from Paenibacillus dendritiformis. with CsgC. The inhibitor proteins were added to CsgA in a substoichiometric ratio of 1:200 and the formation of amyloid was monitored by ThT fluorescence.

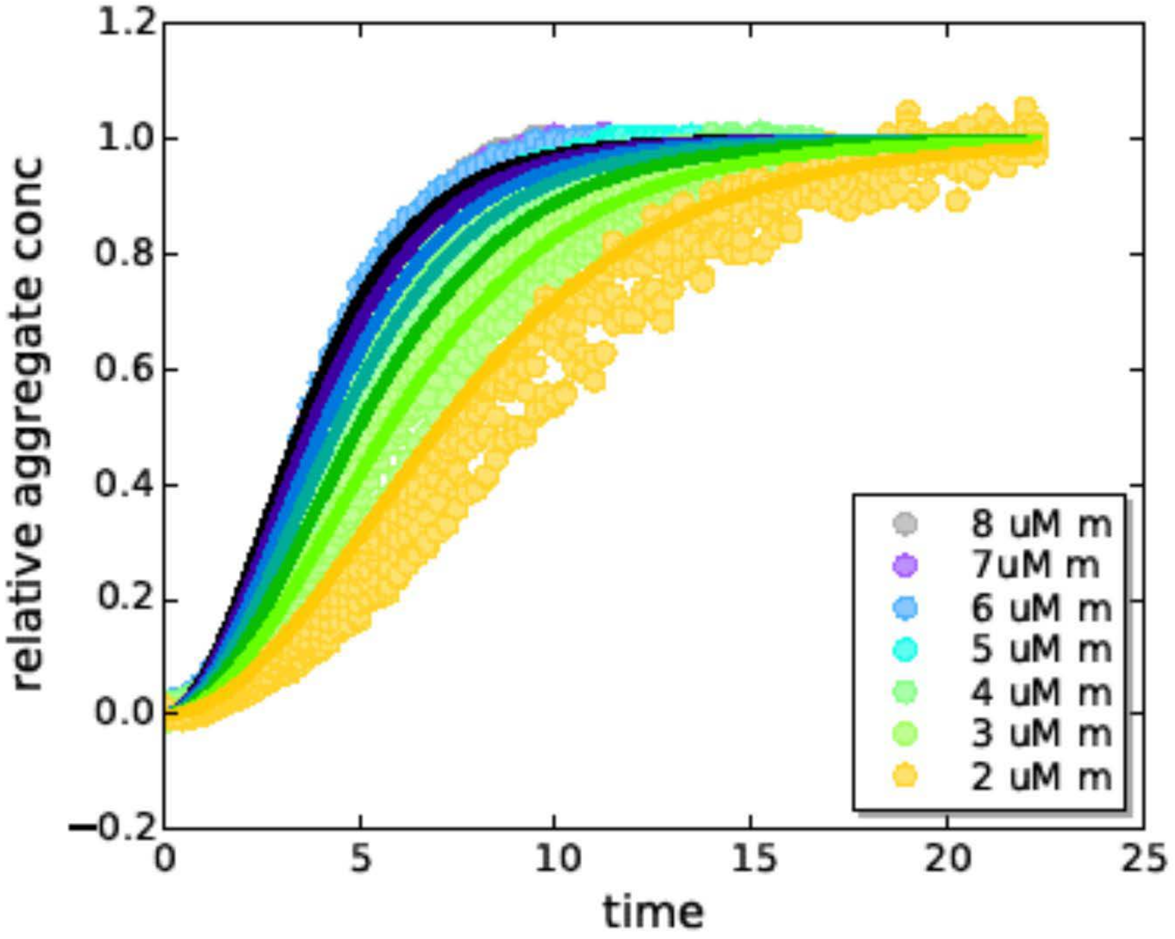

Figure S10. Kinetic analysis of CsgA aggregation in isolation. CsgA at a range of relevant initial CsgA concentrations was allowed to aggregate, and the resultant aggregation curves fitted successfully to an unseeded nucleation-elongation model, containing no secondary processes. The overall rate constant k+kn can be extracted from the fits."

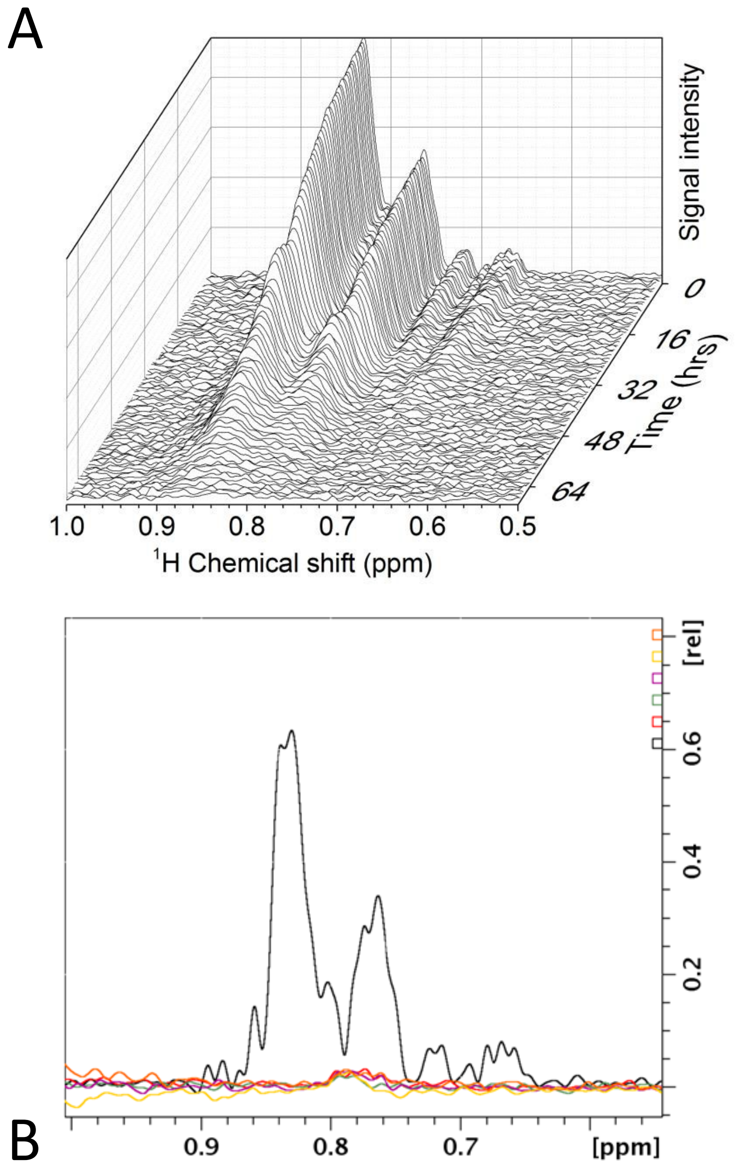

Figure S11. NMR analysis of CsgA aggregation in in the presense of CsgC. (A) NMR spectra of the aggragation 5 µM CsgA shows that CsgA disappears into high molecular weight species in the presence of 1:200 CsgC and there is no evidence medium-sized oligomers remains that would not contribute to THT fluorescence (**B**) NMR spectra of CsgA (+CsgC) extracted at the plateau phase and ultracentrifuged. It shows no detectable protein signal therefore all CsgA has aggregated at plateau.
